# The importance of cancer-associated fibroblasts in targeted therapies and drug resistance in breast cancer

**DOI:** 10.3389/fonc.2023.1333839

**Published:** 2024-01-04

**Authors:** Jian Zheng, Hua Hao

**Affiliations:** Department of Pathology, Yangpu Hospital, School of Medicine, Tongji University, Shanghai, China

**Keywords:** cancer-associated fibroblasts, breast cancer, mechanism, targeted therapy, immunotherapy, drug resistance, prognosis

## Abstract

Cancer-associated fibroblasts (CAFs) play a substantial role in the tumor microenvironment, exhibiting a strong association with the advancement of various types of cancer, including breast, pancreatic, and prostate cancer. CAFs represent the most abundant mesenchymal cell population in breast cancer. Through diverse mechanisms, including the release of cytokines and exosomes, CAFs contribute to the progression of breast cancer by influencing tumor energy metabolism, promoting angiogenesis, impairing immune cell function, and remodeling the extracellular matrix. Moreover, CAFs considerably impact the response to treatment in breast cancer. Consequently, the development of interventions targeting CAFs has emerged as a promising therapeutic approach in the management of breast cancer. This article provides an analysis of the role of CAFs in breast cancer, specifically in relation to diagnosis, treatment, drug resistance, and prognosis. The paper succinctly outlines the diverse mechanisms through which CAFs contribute to the malignant behavior of breast cancer cells, including proliferation, invasion, metastasis, and drug resistance. Furthermore, the article emphasizes the potential of CAFs as valuable tools for early diagnosis, targeted therapy, treatment resistance, and prognosis assessment in breast cancer, thereby offering novel approaches for targeted therapy and overcoming treatment resistance in this disease.

## 1 Introduction

Breast cancer is a prevalent malignancy among women, posing substantial risks to their physical and mental well-being. It is estimated that more than 2.1 million women were newly diagnosed with breast cancer in 2018, including 600,000 deaths and an estimated 2.3 million new cases by 2030 (1, 2). The advancement in patient survival rates can be attributed to the timely identification and enhanced therapeutic interventions, but the efficacy of treatment is hindered by the emergence of metastasis and drug resistance (3, 4). A subset of patients with breast cancer experience a relapse following initial treatment, predominantly in the form of metastatic advanced breast cancer, often accompanied by resistance to chemotherapy (5). Moreover, advanced breast cancer is presently deemed incurable, necessitating the exploration of novel treatment modalities (5). The tumor microenvironment (TME), particularly cancer-associated fibroblasts (CAFs), plays a considerable role in influencing the metastatic potential, recurrence rates, and resistance to treatment in breast cancer (6, 7). Consequently, there has been a growing emphasis on the importance of CAFs in the initiation, progression, invasion, and metastasis of breast cancer, as well as its potential implications for therapeutic interventions.

CAFs represent the most abundant stromal cell population in the TME of breast cancer. Their presence substantially influences the malignant progression and drug resistance of breast cancer. Fibroblasts, which are found in all organs, assume a spindle-shaped morphology during the resting phase and are primarily responsible for shaping the extracellular matrix (ECM) by synthesizing its major constituents and regulating its organization and density (5). Furthermore, their intercellular communication enables them to contribute to the maintenance of tissue integrity (5). Therefore, they determine crucial structural characteristics of the organ, encompassing elasticity, rigidity, and tensile strength. In a state of quiescence or rest, fibroblasts that exist under stable conditions promptly become activated upon disturbance of homeostasis. Research has demonstrated that fibroblasts undergo a metamorphosis in circumstances of inflammation, fibrosis, and wound healing (8–10). Given that both inflammation and fibrosis are linked to the initiation and progression of cancer, fibroblasts are stimulated during these processes (11). These fibroblasts activated in the context of cancer are commonly referred to as CAFs (11). Kojima et al. (12) demonstrated that autocrine transforming growth factor-beta (TGF-β) and stromal cell-derived factor-1 signaling induce the transformation of normal mammary fibroblasts into CAFs. Upon activation, CAFs engage in interactions with tumor cells, thereby facilitating the malignant progression of tumors (13, 14).

CAFs play a crucial role in the construction and remodeling of the ECM, enabling tumor cells to invade the TME and establish interactions with cancer or other stromal cells through the secretion of growth factors, cytokines, and chemokines. These interactions subsequently promote the invasion, progression, metastasis, angiogenesis, immunosuppression, and drug resistance of breast cancer cells (15). Additionally, CAFs can engage in metabolic processes that provide nutritional support for the growth of breast tumors (16). Targeting CAFs is recognized as a potentially effective therapeutic approach owing to their multiple cancer-promoting mechanisms (13, 16). In this paper, we examine the precise mechanism by which CAFs contribute to the initiation and progression of breast cancer. Additionally, the potential importance of CAFs in facilitating early detection, prognostic prediction, treatment resistance and targeted therapeutic interventions for breast cancer is highlighted, thereby providing novel insights into the management of the disease.

## 2 The origin of CAFs

**Figure 1:**
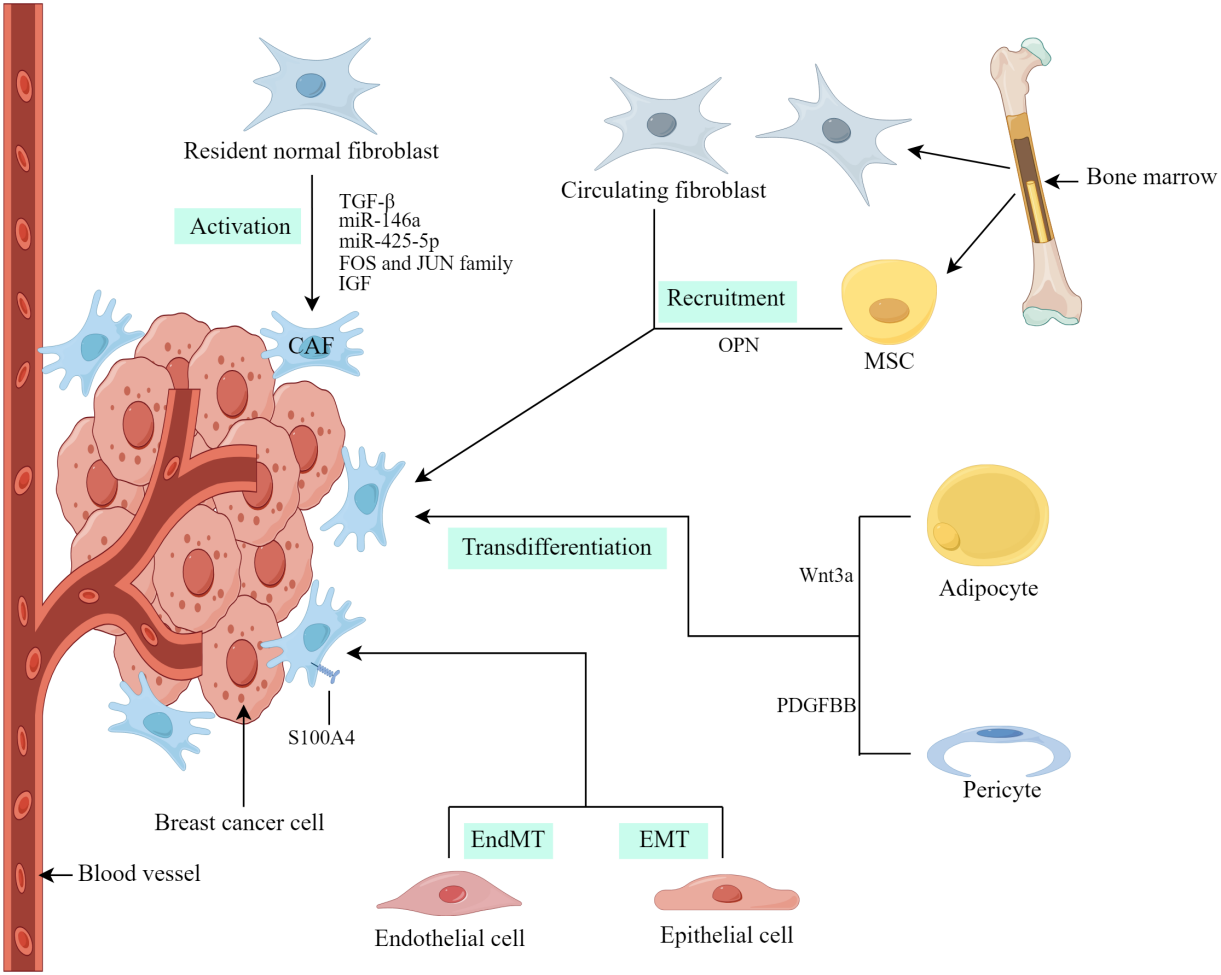
Origins of CAFs in breast cancer. (Created by Figdraw).

At present, the precise origins of CAFs in breast cancer not completely clear. The heterogeneity of CAF characteristics and molecular markers may be due to their different cellular origins (17). In breast cancer, researchers have obtained evidence that CAFs are derived from the origins described below (**Figure 1**).

### 2.1 Resident normal fibroblasts

Resident normal fibroblasts (NFs) within breast cancer are considered a substantial contributor to CAFs and the conversion of NFs into CAFs is influenced by various factors, including growth factors, cytokines, exosomal microRNA (miRNA), and proteins (13). Dysregulation of miRNAs and exosomal miRNAs has been linked to the modulation of CAF formation and activation (18). Yang et al. (19) investigated the regulatory role of breast cancer-derived exosomes in breast cancer cell invasion and metastasis through the action of miR-146a. Additionally, these exosomes were found to accelerate the conversion of NFs to CAFs and promote their recruitment. Zhu et al. (20) observed that miR-425-5p derived from breast cancer exosomes induced the transition of human breast fibroblasts to CAFs through the TGFβ1/ROS signaling pathway. According to Li et al. (21), CAF activation is associated with the enrichment of the FOS and JUN family of transcription factors in activated enhancers, leading to enhanced CAF activation and breast cancer metastasis. De Vincenzo et al. (22) further demonstrated that breast epithelial cells expressing c-Myc paracrine recruit and activate fibroblasts through the insulin-like growth factor (IGF)/IGF-1 receptor axis.

### 2.2 Cells from other sources

CAFs can originate from resident NFs as well as other cell types, including tumor cells with epithelial-mesenchymal transition (EMT), endothelial cells with endothelial-mesenchymal transition (EndMT), pericytes, adipocytes and bone marrow mesenchymal stem cells (MSCs), etc. (5, 17). Weber et al. (23) reported that tumor-derived osteopontin (OPN) induces the transformation of MSCs into CAFs within the microenvironment, thereby promoting tumor growth and metastasis through the OPN–MZF1–TGF-β1 pathway. Furthermore, it has been proposed that fibrocytes, which are mesenchymal progenitor cells derived from circulating bone marrow, may serve as an additional source of CAFs (24). Regarding fibrosis, epithelial cells in close proximity to cancer cells have the potential to undergo EMT and transform into CAFs (25). Therefore, epithelial-derived cancers could potentially harbor a substantial population of CAFs, which play a pivotal role in driving tumor progression. Additionally, endothelial cells have the ability to undergo EndMT and acquire characteristics of CAFs (26). Notably, both transformed epithelial and endothelial cells exhibit the expression of CAF markers, including S100A4 (25, 26). Similarly, Bochet et al. (27) found that Wnt3a secreted by tumor cells triggers the conversion of adipocytes into CAFs in breast cancer by activating the Wnt/β-catenin pathway. These CAFs exhibit elevated expression of fibroblast-specific protein 1 (FSP-1) rather than α-smooth muscle actin (α-SMA) (13). Hosaka et al. (28) presented empirical evidence for vascular pericytes acting as a source of CAFs, considerably facilitating the process of cancer metastasis. The transition from pericytes to CAFs is regulated by the PDGF–BB–PDGFRβ signaling pathway, operating through the mechanism of pericyte-fibroblast transition (28).

## 3 Heterogeneity of CAFs

CAFs exhibit a high degree of heterogeneity and are commonly perceived as dynamic entities that are modified during the initiation and advancement of tumorigenesis rather than being considered an independent cell population (29, 30). The heterogeneity of CAFs is evident in various dimensions, including their origins, subtypes, biomarkers, and physiological roles.

### 3.1 Biomarker heterogeneity of CAFs

The representative markers of CAF include, but are not limited to, α-SMA, serine protease fibroblast activation protein (FAP), FSP-1, PDGFRα, and PDGFRβ. Nevertheless, even α-SMA, which is widely recognized as one of the primary CAF markers, fails to differentiate all CAFs within TME. Additionally, none of these CAF markers exhibit specificity solely to CAFs because they are also expressed in various cell types and healthy tissues (31). Regarding breast and ovarian cancer, four distinct subsets of CAFs, namely, CAF-S1 to CAF-S4, have been identified (13, 32–34). In a subsequent investigation conducted by Kieffer et al. (35), a more comprehensive examination of the FAP+ CAF-S1 subset using single-cell RNA sequencing (scRNA-seq) revealed the existence of eight subclusters. Notably, three of these subclusters exhibited gene expression patterns associated with ECM production and TGF-β signaling and demonstrated a considerable correlation with the presence of CD4+ T cells expressing programmed cell death protein 1 and/or cytotoxic t-lymphocyte-associated protein 4.

Breast cancer can be divided into five molecular subsets based on gene expression. These subtypes include luminal A, luminal B, triple-negative/basal-like, HER2-enriched, and normal-like subtype (36). Jung et al. (37) conducted a microarray analysis to assess the expression levels of various factors, including prolyl 4-hydroxylase, podoplanin, S100A4, chondroitin sulfate proteoglycan (NG2), FAPα, platelet-derived growth factor receptor α (PDGFRα), and PDGFRβ. The results of their study revealed that the adipose interstitial microenvironment was primarily observed in patients diagnosed with luminal A type of cancer, while the fibrointerstitial type was more prevalent among patients with HER-2, luminal B, and triple-negative breast cancers (TNBC) (37). In their study, Park et al. (38) examined the expression profile of various calcium-related proteins in different breast cancer subtypes and discovered that TNBC also exhibited low levels of prolyl 4-hydroxylase, S100A4, and podocin. Additionally, the normal-like stroma displayed limited levels of S100A and prolyl 4-hydroxyl (38).

CAFs encompass a heterogeneous group of cells that exhibit varied responses to stromal stimuli, display distinct secretory phenotypes, and perform specific biological functions within the dynamic TME. The identification of dependable and specific cell surface markers is crucial in discerning different subsets of CAFs (6).

### 3.2 Functional heterogeneity of CAFs

CAFs comprise diverse subsets with distinct functional characteristics, some of which facilitate tumor progression, while others impede it (31, 39). Avalle et al. (40) showed that STAT3 induces breast cancer growth through the secretion of ANGPTL4, MMP13, and STC1 by CAFs. Additionally, Houthuijzen et al. (41) revealed that the CD26+ and CD26-NF populations transform into inflammatory CAFs (iCAFs) and myofibroblast CAFs, respectively. This study further confirmed that CD26+ NF is converted into a pro-tumor iCAF, which recruits bone marrow cells in a CXCL12-dependent manner and enhances tumor cell invasion through the activity of matrix metalloproteinases (MMPs) (41). Jabbari et al. (42) found that CD36+ fibroblasts secrete proteins with dual functions as follows: they impede tumor cell growth by binding to specific receptors as well as enhance the expression of adipogenic markers in CAFs, leading to reprogramming of the tumor matrix. Additionally, the findings of Barone et al. indicated that the activation of the nuclear Farnesoid X Receptor hampers the tumor-promoting capabilities of CAFs by influencing their mechanical characteristics and paracrine signaling repertoire (43).

### 3.3 Temporal heterogeneity of CAFs

Venning et al. (44) developed a multicolor flow cytometry strategy based on the exclusion of non-CAFs and successfully used this strategy to explore the temporal heterogeneity of freshly isolated CAFs in 4T1 and 4T07 mouse models of TNBC. This study found that the expression of six CAF markers, α-SMA, FAPα, PDGFRα, PDGFRβ, CD26, and PDPN, all changed over time as tumors matured, from predominantly PDGFRα+ fibroblasts in healthy breast tissue to predominantly PDGFRβ+ CAFs in tumors. The abundance and dynamics of each marker varied depending on tumor type and time, providing evidence for temporal coevolution of CAF populations (44).

Furthermore, the heterogeneity of tumors is influenced by the inherent evolution of individual subclones as well as the selective pressures exerted by the surrounding environment (45). The TME encompasses various components, such as peripheral blood vessels, immune and inflammatory cells, fibroblasts, ECM, and signaling molecules, all of which engage in continuous and active communication with cancer cells (46). Consequently, the microenvironment has a positive contribution to tumor heterogeneity (47). Among these components, CAFs, being a crucial element of the TME, exhibit substantial heterogeneity and may potentially impact tumor heterogeneity. Li et al. (48) comprehensively characterized cervical cancer cell heterogeneity using scRNAseq and identified epithelial cells, fibroblasts, and CD8+ T cell subsets, illustrating the cellular heterogeneity of cervical cancer, suggesting that tumor fibroblasts contribute to cervical cancer progression. Chen et al. (49) employed scRNAseq to investigate the evolving dynamics of the TME of pancreatic ductal carcinoma (PDAC). Through this analysis, the researchers discovered a distinct population of CAFs termed complement-secreted CAFs (csCAFs), which selectively express a subset of complement system components. Additionally, the team utilized weighted gene co-expression network analysis to construct modules associated with csCAFs. Notably, csCAFs were found exclusively in early stages of PDAC and were localized in close proximity to malignant cells within the surrounding tissue matrix (49). The investigation of microenvironment heterogeneity has been limited, yet it is evident that CAFs encompass a diverse population, the extent of which is not completely understood regarding their influence on tumor heterogeneity (50).

## 4 The role and mechanism of CAFs in breast cancer

### 4.1 The role of CAFs in proliferation, migration, and invasion of breast cancer

**Figure 2:**
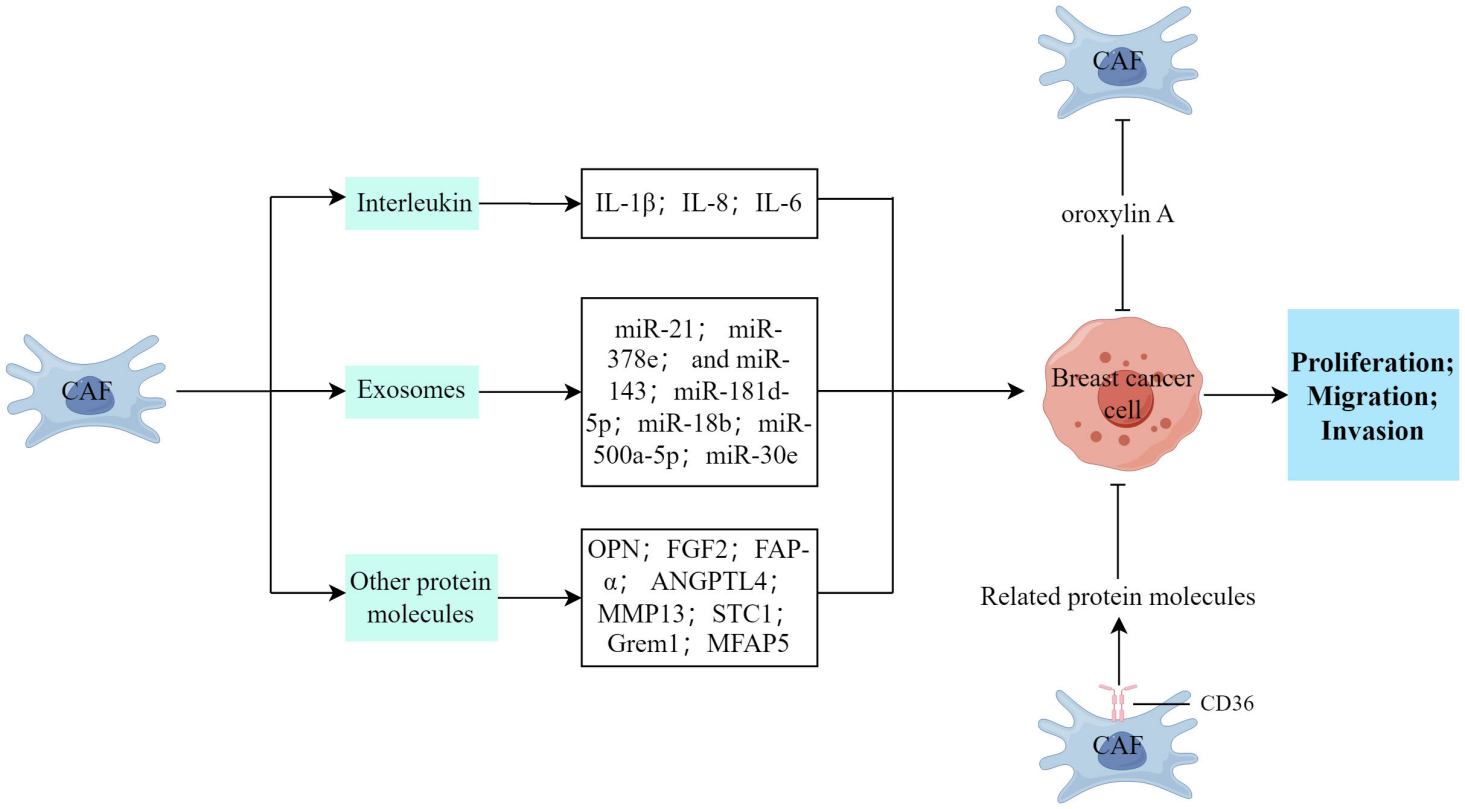
The impact of CAFs on the proliferation, invasion, and metastasis processes of breast cancer cells. (Created by Figdraw).

As shown in **Figure 2**, CAFs in breast cancer can enhance the proliferation, invasion, and metastasis of breast cancer cells. This section primarily provides a summary of the involvement of diverse cytokines, protein molecules, and exosomes released by CAFs in the malignant advancement of breast cancer.

#### 4.1.1 CAFs and interleukin

The secretion of interleukin (IL) by CAFs is substantially associated with cancer development. Ershaid et al. (51) discovered that the secretion of IL-1β by CAFs plays a crucial role in promoting breast cancer metastasis to the lungs through various mechanisms. These include the modulation of the immune cell environment at the metastatic site and the upregulation of adhesion molecules at both the primary tumor and the metastatic site, and these processes potentially enhance the invasion and dissemination of tumor cells (51). Santolla et al. (52) demonstrated that the paracrine signaling of IL-8/CXCR1/2 could effectively activate and induce the migration and invasion properties of MDA-MB-231 breast cancer cells. Similarly, Sun et al. (53) discovered that IL-6 exerted a downregulating effect on the tumor suppressor, HIC1, thereby facilitating the development of breast cancer within the TME through paracrine or autocrine signaling mechanisms.

#### 4.1.2 CAFs and exosomes

The exosome signaling pathway, which involves the presence of miRNA, serves as a means of communication between CAFs and tumors and CAF-released exosomes can transfer to breast cancer cells, releasing pertinent information and targeting specific genes (18). Additionally, CAFs can secrete exosomes that contain a high concentration of miR-21, miR-378e, and miR-143 which play a considerable role in augmenting the stemness of tumors, modifying the EMT phenotype, and facilitating the proliferation and metastasis of tumors (19, 54). CAFs can release exosomes that carry miR-181d-5p, facilitating the processes of EMT, proliferation, migration, and invasion in breast cancer cells. This is achieved by specifically targeting caudal-type homeobox 2 and subsequent downregulation of homeobox A5 (55). Furthermore, Yan et al. (56) discovered that exosomes containing miR-18b, derived from CAFs, facilitated aberrant nuclear Snail expression by targeting TCEAL7, thereby activating the nuclear factor kappa B (NF-κB) pathway and subsequently inducing EMT, invasion, and metastasis in breast cancer. Similarly, Chen et al. (57) found that exosomal miR-500a-5p, originating from CAFs, enhanced breast cancer cell proliferation and metastasis by targeting USP28. Moreover, the activation of FAK signaling in CAFs facilitates the migration and metastasis of breast cancer cells through intercellular communication mediated by exosomal miRNAs (58). However, Xi et al. (59) discovered that the upregulation of miR-30e or the downregulation of CTHRC1 impeded the proliferation and migration/invasion of breast cancer cells and stimulated apoptosis.

#### 4.1.3 CAFs and other protein molecules

CAFs can facilitate the infiltration and spread of breast cancer cells through the secretion of various cytokines and protein molecules. Muchlińska et al. (60) observed that α-SMA-positive CAFs potentially contribute to tumor expansion by releasing OPN. Additionally, CAFs stimulate the proliferation, migration, and invasion of MDA‐MB‐231 cells within the breast TME through the paracrine FGF2–FGFR1 signaling pathway (61). Huang et al. (62) demonstrated that TGF-β1-activated CAFs facilitated tumor invasion, lung metastasis, and EMT through autophagy and FAP-α overexpression in both experimental models. Conversely, the effects induced by TGF-β1-activated CAFs were hindered by the autophagy inhibitor, 3-methyladenine (62). Avalle et al. (40) provided evidence that STAT3 plays a crucial role in the pro-tumorigenic functions of murine CAFs in breast cancer, both *in vitro* and *in vivo*. This is achieved through the secretion of ANGPTL4, MMP13, and STC1 by CAFs, which promotes breast cancer growth. Ershaid et al. (51) established a connection between tissue damage, inflammation, and the progression and metastasis of breast cancer. They identified the involvement of the NLRP3 inflammasome in fibroblasts, specifically in CAFs, in mediating tumor growth and facilitating the recruitment of CD11b+Gr1+ bone marrow cells into the TME (51). Ren et al. (63) explored the autocrine role of Grem1, which is produced by CAFs, in promoting fibroblast activation. Additionally, they demonstrated the paracrine effect of Grem1 in stimulating breast cancer cell stemness and invasion. Another study reported that interfering with prostaglandin E2 signaling in CAFs inhibits mammary carcinoma growth, but enhances metastasis (64). Furthermore, Chen et al. (65) discovered that CAFs facilitate cancer cell invasion and migration by secreting MFAP5 and activating the Notch1/slug signaling pathway.

#### 4.1.4 The inhibitory effect of CAFs on proliferation and metastasis of breast cancer

Jabbari et al. (42) discovered that the proteins secreted by CD36+ fibroblasts inhibited the growth of tumor cells by binding to their corresponding receptors and enhanced the expression of adipogenic markers in CAFs, thereby reprogramming the tumor stroma. Similarly, Cao et al. (66) observed that oroxylin A deactivated CAFs and suppressed breast cancer metastasis by selectively binding to ACTN1 and inhibiting its expression.

### 4.2 CAFs and tumor angiogenesis

**Figure 3:**
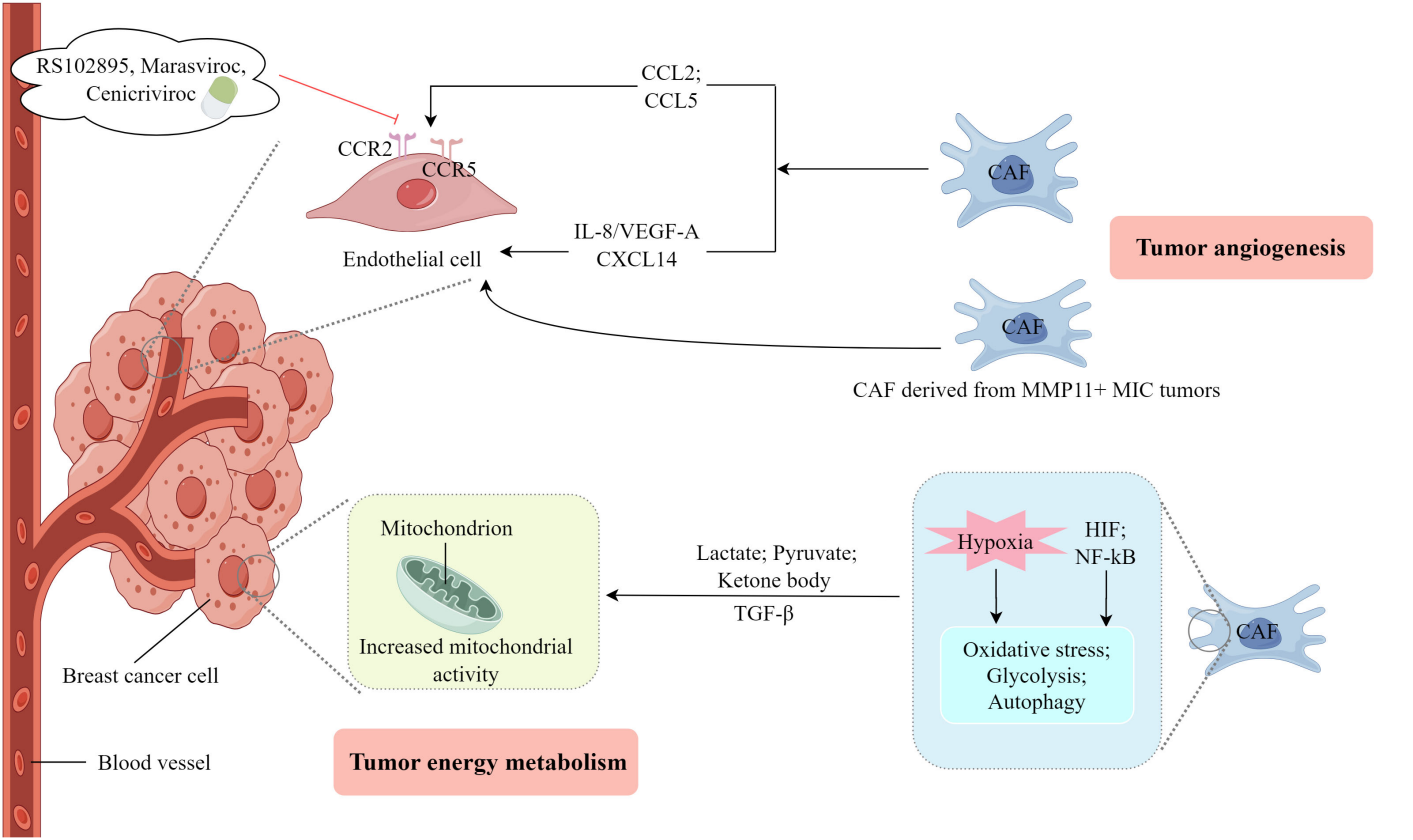
The involvement of CAFs in the process of tumor angiogenesis and energy metabolism. (Created by Figdraw).

CAFs facilitate the processes of angiogenesis and lymphangiogenesis as well as provide essential nourishment for the growth and invasion of tumors (67, 68). As shown in **Figure 3**, CAFs secrete vascular endothelial growth factors (VEGFs) and release various signaling molecules that stimulate endothelial cells, thereby initiating angiogenesis (69). Al-kharashi et al. (70) discovered that CAFs in breast cancer exhibiting high levels of DNA methyltransferase 1 could enhance the expression of IL-8/VEGF-A, thereby facilitating angiogenesis, a process strongly associated with the unfavorable survival outcomes of patients with breast cancer. Similarly, Wan et al. (71) observed that CAFs in breast cancer characterized by elevated levels of FOS-like antigen 2 could stimulate the sprouting of human umbilical vein endothelial cells independent of VEGF, leading to angiogenesis and tumor growth *in vivo*. CAFs exhibiting increased expression of the epithelial chemokine CXCL14 induce EMT in breast cancer cells, thereby facilitating their migration and invasion (72). This process depends on the presence of nitric oxide synthase-1 and encompasses the activation of angiogenesis and the recruitment of macrophages (73). Eiro et al. found that CAFs, particularly those derived from MMP11+ MIC tumors, could promote breast cancer cell invasion and angiogenesis (74). Furthermore, substantially upregulated long non-coding RNA (lncRNAs), *SNHG5*, and its downstream signaling molecule, ZNF281CCL2/CCL5, in CAFs play a pivotal role in establishing the premetastatic niche in breast cancer (75). These molecules also influence angiogenesis and vascular leakage by modulating the activity of ZNF281 in CAFs. Additionally, the inhibitors, RS102895, marasviroc, and cenicriviroc effectively impede angiogenesis and vascular permeability within the premetastatic niche by obstructing the binding of CCL2/CCR2 and CCL5/CCR5 (75).

### 4.3 CAFs and tumor energy metabolism

An energy metabolism coupling relationship exists between CAFs and breast cancer cells (76). As shown in **Figure 3**, lactate produced by hypoxic CAFs serves as a metabolic mediator that facilitates the growth and invasion of breast cancer cells. This is achieved by activating the TGFβ1/p38 MAPK/MMP2/9 signaling pathway and enhancing mitochondrial activity in cancer cells (77). Therefore, targeting oxidized ataxia-telangiectasia mutated could be a viable therapeutic approach for breast cancer (77). The heightened metabolic activity observed in neoplastic cells results in elevated glucose absorption, glycolysis, and lactic acid generation which prompts CAFs to secrete TGF-β, consequently inducing a shortage of phosphoenolpyruvate. This metabolic deficiency hampers the functionality of activated T cells and promotes the conversion of CD4+T cells into T helper 2 cells (78, 79). CAFs facilitate the transfer of substrates (e.g. lactate, pyruvate, and ketone bodies) to neighboring cancer cells through an autophagy-mediated paracrine mechanism, where these substrates originate from the increased glycolytic metabolism of CAFs (80). Several studies have indicated the existence of metabolic coupling between catabolic fibroblasts and anabolic cancer cells in breast cancer, prostate cancer, head and neck cancer, and lymphoma. This metabolic coupling is responsible for driving oxidative stress, glycolysis, autophagy, and senescence of fibroblasts through the activation of hypoxia-inducible factor and NF-κB signaling pathways (81). The fibroblast breakdown and resultant metabolic byproducts create a microenvironment abundant in nutrients that supports the growth of tumors. This is achieved by producing mitochondrial fuel, including lactate, ketone bodies, fatty acids, glutamine, and various amino acids, within the nearby ECM (81). Autophagy is presumed to have an anti-tumor effect on normal tissues and early tumors, but a pro-tumor effect on established cancer cells (82). These studies indicate that CAFs substantially impact the invasion and progression of breast cancer cells by engaging in various metabolic coupling pathways, and the findings potentially provide novel therapeutic approaches to manage breast cancer.

### 4.4 CAFs and TME

The TME comprises diverse cellular components and ECM, which are closely associated with the development, progression, metastasis, and prognosis of breast tumors.

#### 4.4.1 Remodeling of the ECM

**Figure 4:**
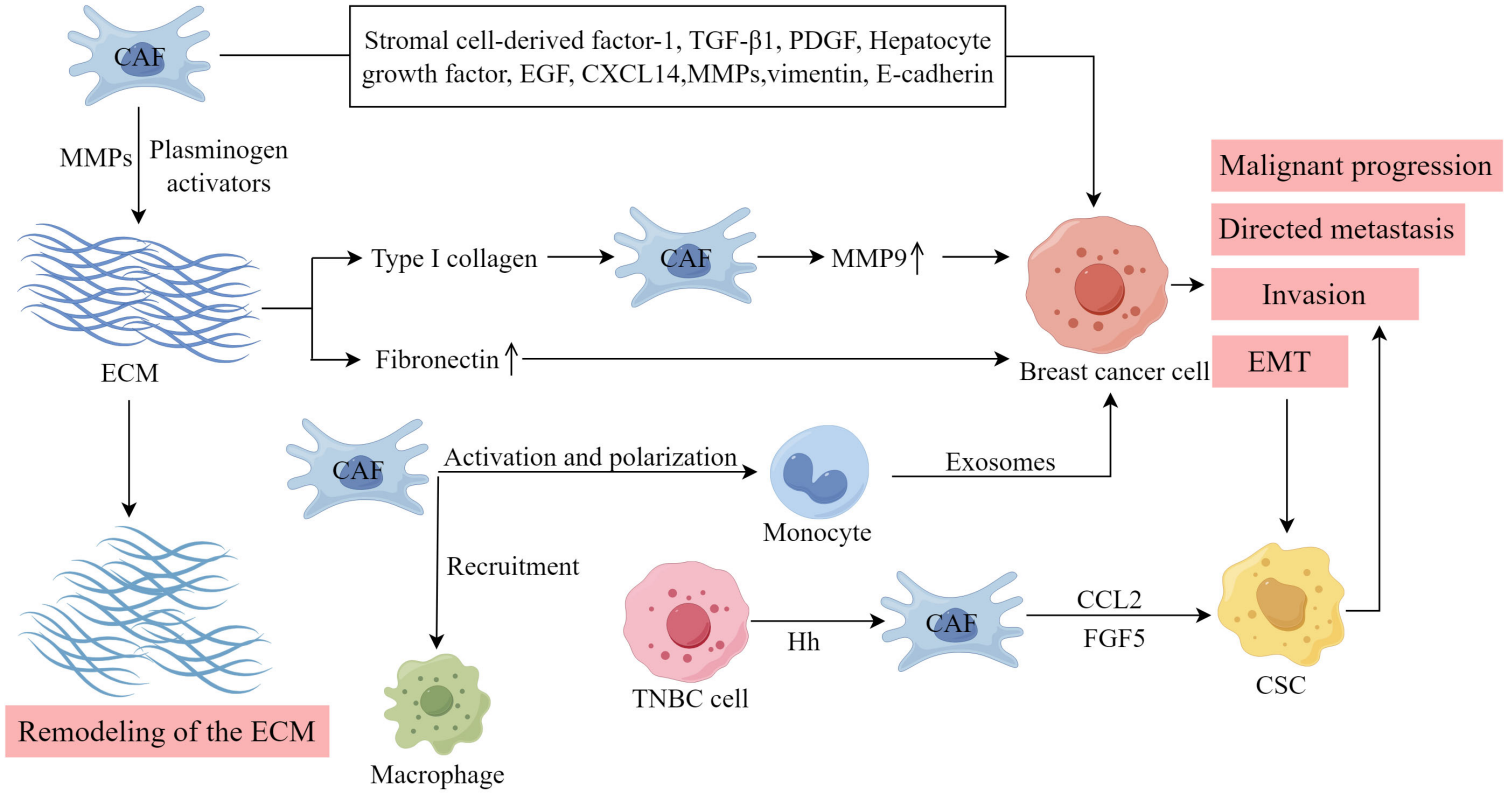
The importance of CAFs within the TME. (Created by Figdraw).

An aberrant production and remodeling of the ECM is a distinctive feature of CAFs. As shown in **Figure 4**, in breast tumors, type I collagen is recognized as the principal constituent of the ECM, and its presence is associated with tumor cell survival and the occurrence of metastasis. Type I collagen fibers play a vital role in stimulating the expression of MMP-9 in CAFs in breast cancer, thereby facilitating increased migration and metastasis (83). Notably, the overexpression of MMP-9 in breast cancer cell lines by CAFs substantially amplifies tumor invasiveness, primarily by activating the TGF-β/SMAD signaling pathway (84). CAFs can generate various types of MMPs and plasminogen activators, which directly degrade the ECM. Consequently, this process facilitates the invasion and metastasis of breast cancer cells. Additionally, CAFs create a migratory route for tumor cells, thereby promoting their infiltration into the blood and lymphatic system (85). Fibronectin (FN) is an important ECM molecule found in various malignant tumors, influencing tumor cell proliferation, migration, EMT, and angiogenesis (86, 87). The activation of CAFs can stimulate the upregulation of FN expression and alter the organization of FN fibers, thereby facilitating the directed migration of tumor cells (88). Consequently, CAFs play a crucial role in the process of ECM remodeling, facilitating the synthesis and remodeling of the ECM in breast cancer through various pathways, thereby promoting tumor growth.

#### 4.4.2 The secretion of a variety of cytokines and protein molecules

CAFs exert regulatory control over tumor cells and the TME by secretion of growth factors, cytokines, and chemokines (13). Stromal cell-derived factor-1, TGF-β1, PDGF, hepatocyte growth factor, epidermal growth factor, and MMP are involved in the induction of EMT in breast cancer by CAFs (89, 90). Furthermore, chemokines, proteins ranging from 8 to 14 kDa, were identified as CAF-secreted factors associated with EMT in tumor cells. These chemokines stimulate directional cell migration by establishing a gradient that guides the movement of cell types expressing the corresponding receptor (91). CAFs with upregulated epithelial chemokine CXCL14 induce EMT in breast cancer and facilitate the migration and infiltration of breast cancer cells (72). This process depends on the availability of nitric oxide synthase-1 and encompasses the activation of angiogenesis and the recruitment of macrophages (73). EMT imparts mesenchymal properties to epithelial cells, which is strongly associated with the invasive traits of the cancer stem cell (CSC) phenotype. CAFs in breast cancer can also induce EMT by upregulating vimentin and downregulating E-cadherin (92). Breast CSCs are distinguished by their CD44+/CD24- phenotype and can generate circulating tumor cells, thereby facilitating tumor invasion and metastasis. CAFs enhance the expression of the cytokine CCL2 by activating the NOTCH1-STAT3 pathway, and CCL2 subsequently promotes the development of the CSC phenotype. In an animal model of breast cancer, inhibiting CCL2 production by fibroblasts effectively suppressed tumor formation (93). Furthermore, TNBC cells can secrete Hh factors. This interaction activates the SMO pathway upon binding to receptors on neighboring CAFs, thereby facilitating the co-secretion of FGF5 within the cells. This process plays a crucial role in maintaining the stemness of tumor cells (94).

#### 4.4.3 Cell-cell interactions

Liu et al. discovered that extracellular ATP facilitated the interactions between fibroblasts and breast cancer cells, leading to a collaborative production of S100A4, which further exacerbated breast cancer metastasis (95). Pakravan et al. observed that monocytes, once activated by CAFs and polarized, experienced a decline in their ability to eliminate tumors. Furthermore, the exosomes derived from these monocytes facilitated the proliferation and migration of breast cancer cells. Conversely, exosomes from CAF-educated monocytes demonstrated a substantial enhancement in breast cancer tumorigenicity *in vivo* (96).

### 4.5 CAFs and immune regulation

**Figure 5:**
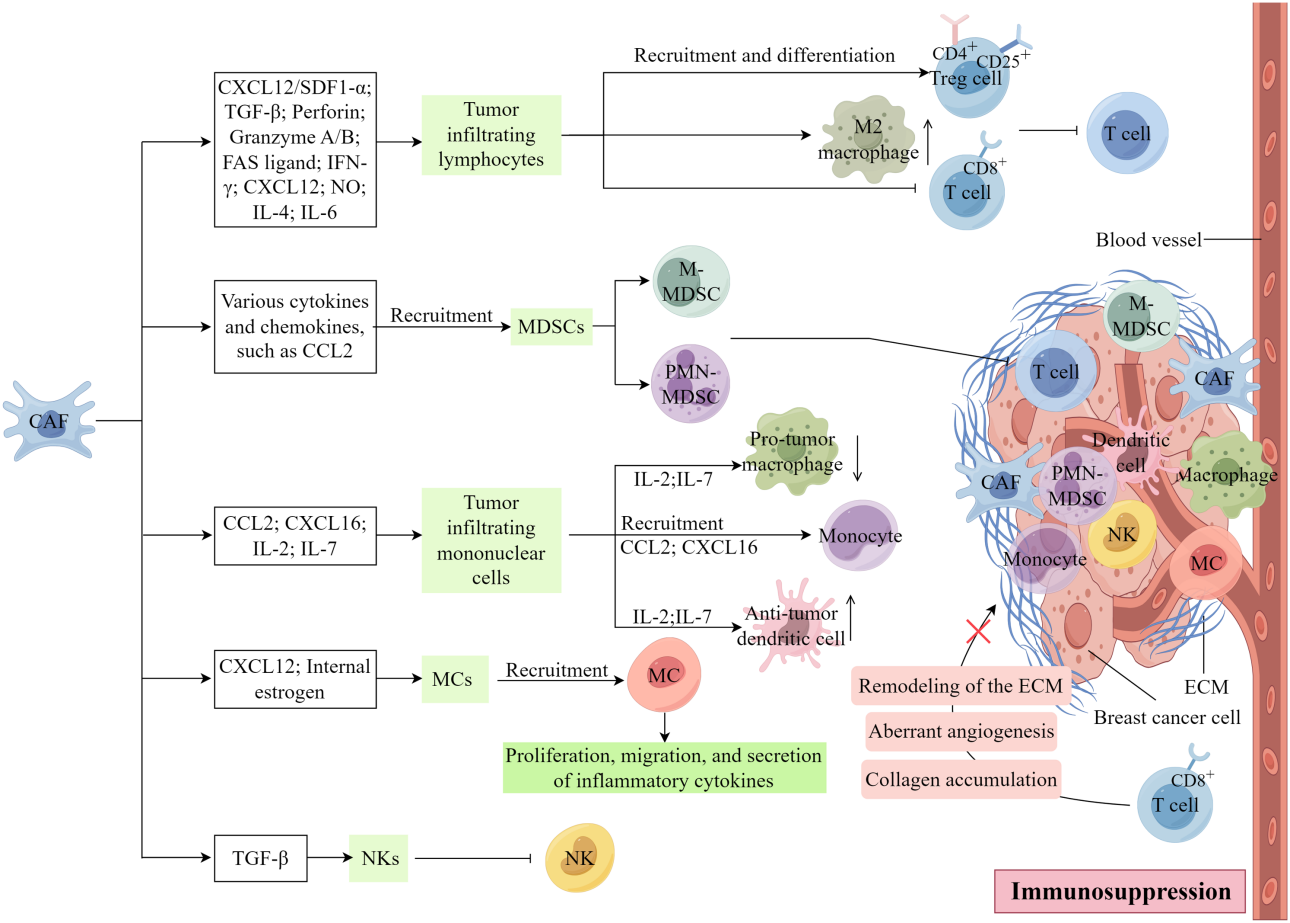
The involvement of CAFs in the regulation of tumor immunity. (Created by Figdraw).

CAFs exert a direct or paracrine influence on immune cell function or impede physical interactions between immune and cancer cells. Consequently, this diminishes the ability of the immune system to recognize and eliminate cancer cells, thereby facilitating tumor immune evasion, a critical mechanism in tumor progression (13, 39). As shown in **Figure 5**, CAFs substantially associate with the inhibition of immune cells within the TME.

#### 4.5.1 Tumor infiltrating lymphocytes

The tumor infiltrating lymphocytes (TILs) found within tumor tissues are characterized by substantial heterogeneity and serve as integral components in the host’s immune response against tumor-specific antigens. Within various cancerous tissues, TILs exhibit distinct properties, either promoting or inhibiting tumor growth, contingent upon the specific T-cell subset involved (3). The elimination of FAP+ CAFs *in vivo* induce a shift in the immune microenvironment from Th2 polarization to Th1 polarization, indicating that CAFs may serve as promising targets for metastatic breast cancer (97). Furthermore, the CAFs-s1 subtype in breast cancer enhances the recruitment and differentiation of CD4+CD25+ Treg in the TME through the CXCL12/SDF1-α pathway, thereby suppressing the function of effector T cells (33). α-SMA+ CAFs have been identified as an important cellular source of TGF-β, which hampers the cytotoxic activity of CD8+ T cells by downregulating the expression of perforin, granzyme A/B, FASL (FAS ligand), and IFN-γ (98, 99).

CAFs can impede the proliferation of effector T cells and recruit CD4+ CD25+ T cells to the tumor stroma by secreting CXC chemokine ligand 12, and this recruitment leads to the transformation of CD4+ CD25+ T cells into CD25+ forkhead box protein 3+ T cells, inducing immunosuppression and drug resistance within the TME of TNBC. However, CAFs also impede the proliferation of CD4+CD8+ T cells by releasing nitric oxide. Additionally, CAFs can augment the population of M2 macrophages by secreting IL-4 and IL-6, consequently hindering T-cell functionality (33, 100–102). CAFs can additionally attract various immune cells, including macrophages, neutrophils, and T cells, towards the neighboring stroma. Consequently, these immune cells are hindered from infiltrating the cancerous tissue and executing their typical anti-tumor immune response (78).

#### 4.5.2 Myeloid-derived suppressor cells

Myeloid-derived suppressor cells (MDSCs) encompass a diverse assemblage of myeloid cells that contribute to immunosuppression through the inhibition of cytokines and other molecules, thus facilitating the advancement and metastasis of cancer (3). Two primary MSDC subsets exist, namely polymorphonuclear MDSCs (PMN-MDSCs) and monocytic MDSCs (M-MDSCs), which are similar to neutrophil and monocyte phenotypic and morphological characteristics, respectively (103, 104). A novel subgroup of MDSCs, known as circulating fibroblasts, demonstrates phenotypic and functional similarities to CAFs, implying a potential correlation between MDSCs and CAFs (105). CAFs, by releasing various cytokines and chemokines, have the capability to facilitate the infiltration and generation of MDSCs, thereby impeding the antitumor efficacy of effector T cells. Evidence suggests that CCL2 plays a crucial role in the recruitment of both PMN-MDSCs and M-MDSCs (106, 107). As the primary source of CCL2, CAFs may induce the migration of MDSCs to the tumor site through the activation of STAT3 signaling pathways (108).

#### 4.5.3 Tumor infiltrating mononuclear cells

Monocytic myeloid cells, encompassing monocytes, terminally differentiated macrophages, and dendritic cells, constitute a diverse population of bone marrow-derived cells (3). These myeloid cells play a crucial role in tumor progression by engaging in direct interactions with tumor cells or providing support to the tumor stroma, thereby facilitating tumor growth, angiogenesis, migration, invasion, metastasis, and suppression of tumor immunity (109, 110). CAFs are capable of attracting monocytes to breast tumors through the secretion of CCL2 and CXCL16 (111, 112). *In vitro* experiments have demonstrated that the inhibition of IL-6 leads to a reduction in CCL2 secretion and subsequent recruitment of monocytes (112). In their study utilizing a 4T1 mouse metastatic breast cancer model, Liao et al. (97) provided evidence that the absence of FAP+ CAFs led to heightened levels of IL-2 and IL-7 expression, while dampening the expression of IL-6, IL-4, VEGF, and CSF-1. Consequently, this alteration resulted in a diminished influx of pro-tumor macrophages and regulatory T cells (Tregs), alongside an augmented recruitment of anti-tumor dendritic and cytotoxic T cells (97).

#### 4.5.4 Mast cells and natural killer cells

The activation of CAFs has been observed through the release of IL-13 and trypsin by MCs (113). Notably, MCs, once activated contribute to tumor progression and immunity. CAFs play a role in tumorigenesis by promoting the proliferation, migration, and secretion of inflammatory cytokines in mast cells through the upregulation of internal estrogen in prostate cancer (114). Additionally, estrogen-induced CAFs contribute to the recruitment of MCs through the production of CXCL12 and the activation of CXCR4 (114). Furthermore, a recent study in a microtissue model of prostate cancer demonstrated a correlation between MCs and CAFs to induce an early malignant morphological transformation of benign epithelial cells (115). To date, studies on the correlation between MCs and CAFs in tumors are still lacking.

Additionally, α-SMA+ CAFs, serving as the primary origin of TGF-β, possess the ability to modulate the functionality of NKs (6, 102). Numerous studies have highlighted the role of TGF-β in suppressing NK activation and the importance of their cytotoxic activity (116). For instance, the induction of miR-183 by TGF-β hinders the transcription of DAP12 and reduces the expression of the activating receptors, NKp30 and NK Group 2D (NKG2D), in NKs, consequently impeding their cytotoxicity (117).

Furthermore, the presence of various surface molecules on CAFs, including dipeptidopeptidase 4, junctional adhesion molecule 2, immune checkpoint B7-H3, and calbindin 11, facilitate immune escape within the TME. These molecules play a crucial role in mediating immune cell migration, proliferation, and differentiation (33, 78). Zheng et al. discovered that secreted disaccharide chain proteoglycans played a role as tumor promoters and immunosuppressors in TNBC, specifically within the context of CAFs (118). Furthermore aberrant angiogenesis, collagen accumulation, and the remodeling of the ECM collaborate to reconstruct the tumor stroma, resulting in the formation of a rigid and compact barrier encasing the cancerous cluster. This formidable structure impedes the infiltration and subsequent assault of CD8+ T cells on the malignant cells (100, 119). Therefore, CAFs can change the immune function of immune cells in the TME in different ways, affect the immune response, and lead to immunosuppression.

## 5 The role of CAFs in the diagnosis of breast cancer

Given the considerable contribution of CAFs to breast cancer progression and their specific association with tumors, researchers have attempted to assess their potential for on-time diagnosis of breast cancer. Giussani et al. (120) conducted a study where they observed elevated plasma levels of type IX and X collagen α1 and cartilage ligament matrix in patients with breast cancer compared to those with benign lesions and healthy individuals. Furthermore, *in vitro* experiments demonstrated increased expression of these proteins in fibroblasts cultured with tumor cell-conditioned medium (120). Thus, it was suggested that the expression of these proteins by fibroblasts could serve as reliable biomarkers for distinguishing between benign and malignant tumors (120). Notably, Yamaguchi et al. (121) categorized patients with invasive breast cancer into PDPN-positive and -negative groups based on the presence of PDPN-positive CAF and reported its relationship with magnetic resonance imaging findings. Invasive breast cancer in PDPN-positive CAF tended to have a more malignant pathological state (121). Pelon et al. (34) discovered that the accumulation of CAF subsets in the lymph nodes (LN) serves as a prognostic indicator, thereby indicating the potential examination of CAF subsets in axillary LN during the initial diagnosis. Additionally, several studies have demonstrated the frequent detection of increased levels of Wnt5a in the serum of patients with breast cancer, which exhibited a strong correlation with microvessel density in breast tumor tissues (71). This correlation suggests the potential clinical importance of Wnt5a as a diagnostic tool for breast cancer (71).

Despite the high detectability of most markers found in CAFs, their specificity remains limited. Consequently, recent advancements in genetic testing techniques have facilitated investigations into the gene expression profile of CAFs in breast cancer. Hang et al. (122) compared the gene expression profiles between CAFs derived from primary breast malignant tumors and normal breast stromal cells and successfully identified eight key genes that exhibited differential expression through this analysis. These findings have the potential to identify patients with breast cancer with a poor prognosis (122).

## 6 The role of CAFs in breast cancer treatment and drug resistance

Currently, breast cancer management primarily depends on surgical resection, complemented by radiotherapy, chemotherapy, endocrine therapy, and immunotherapy. Despite substantial advancements in breast cancer treatment, the challenges of tumor recurrence and drug resistance persist as major concerns (3, 4). Numerous anti-cancer modalities, including targeted therapy, chemotherapy, radiotherapy, and immunotherapy, have demonstrated the potential to reduce tumor size and facilitate remission in certain patients. Nevertheless, the emergence of resistance among tumor cells towards these therapeutic interventions stimulates persistent tumor cell proliferation. Consequently, understanding the underlying mechanisms of drug resistance in tumor cells to identify novel and more efficacious treatment approaches for breast cancer is essential.

### 6.1 CAFs as therapeutic targets for breast cancer

**Figure 6:**
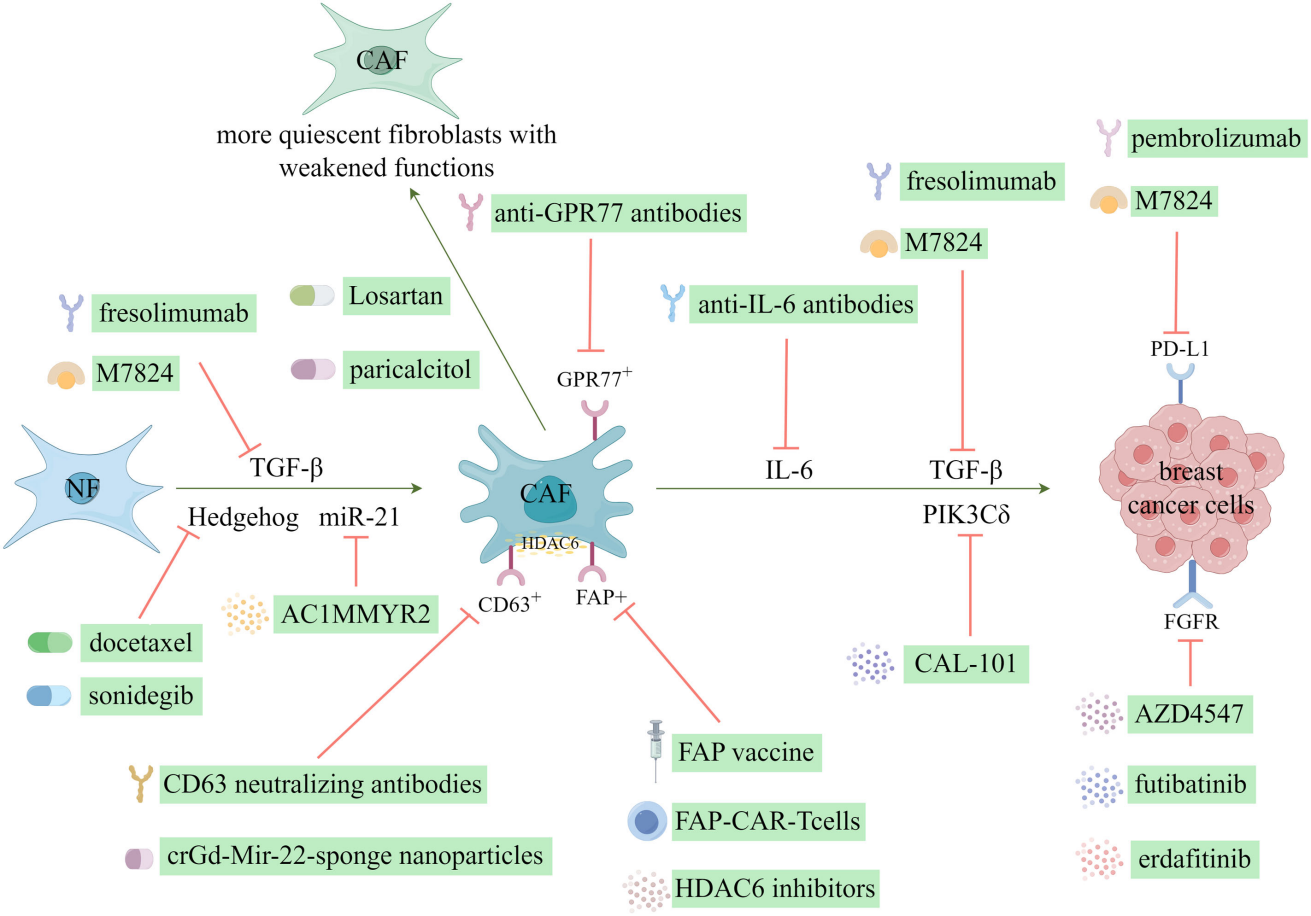
CAF-targeting therapeutic strategies in breast cancer. (Created by Figdraw).

Currently, various CAF-mediated anticancer therapies exist, most of which are in the preclinical trial phases. Generally, these therapies can be categorized into the following five application approaches: hindering the transition from NFs to CAFs, facilitating the reversion from CAFs to NFs, impeding tumor growth and advancement, stimulating the immune system, and reversing tumor chemoresistance (123). Notably, breast cancer has been extensively investigated as a primary focus for targeting CAFs in cancer treatment as shown in **Figure 6**.

#### 6.1.1 Targeting the origin of CAFs or reversion to a non-CAFs state

The activation of resident fibroblasts plays a pivotal role in forming CAFs and has been recognized as an important target for therapeutic interventions in breast cancer studies. Cazet et al. (124) conducted a study on a TNBC model, where they discovered that the activation of Hedgehog-dependent CAFs and the remodeling of the ECM contribute to the development of CSC niches, ultimately leading to resistance against the chemotherapeutic agent, docetaxel. They proposed therapeutic interventions targeting this pathway to address this issue, which yielded promising outcomes in preclinical models (13, 124). Consequently, these positive results prompted the initiation of phase I and II clinical trials involving the combination of the smoothened inhibitor, sonidegib, with docetaxel (13). Losartan, which is an angiotensin inhibitor, demonstrated the inhibition of angiotensin-II receptor-1 in CAFs, resulting in decreased expression of downstream signaling molecules, including TGF-β, connective tissue growth factor, and endothelin-1 (125). This inhibition led to a reduction in stromal collagen and hyaluronan production within tumors and an enhancement in drug and oxygen delivery (125). Additionally, Coulson et al. (126) demonstrated the efficacy of losartan in impeding mammary tumor development and progression in a murine model. Furthermore, scientific evidence has shown the efficacy of all-trans retinoic acid in inducing a transformation of CAFs into a state of reduced activity, thereby effectively suppressing their biological functions (13). Additionally, paricalcitol, which is a vitamin D receptor agonist, possesses the ability to deactivate CAFs (127). Certain miRNAs can activate fibroblasts and induce their transformation into CAFs. Conversely, the expression of miR-21 facilitates the reversion of CAFs into NFs, thereby effectively enabling the remodeling of CAFs (128). AC1MMYR2, which is a small molecule inhibitor of miR-21, functions by upregulating the miR-21 target protein, Von Hippel-Lindau, consequently reducing the activity of the NF-κB pathway (128). This inhibition of miR-21 and subsequent modulation of the NF-κB pathway attenuates CAF-induced EMT and enhances the responsiveness to paclitaxel (128).

#### 6.1.2 Targeting CAFs related protein molecules and signaling pathways

The interaction between CAFs and neighboring tumor cells, as well as the TME, through protein secretion, has led to the identification of these proteins and their associated signaling pathways as promising targets for therapeutic intervention. TGF-β, as a crucial mediator of the reciprocal interactions between CAFs and breast cancer cells, represents an important therapeutic target expressed in CAFs. Preclinical investigations have shown that small-molecule inhibitors directed towards TGF-β receptors effectively impede the aggressive behavior of breast cancer cells (13, 127, 129, 130). Furthermore, clinical trials have commenced for neutralizing antibodies that specifically target TGF-β. One such monoclonal antibody, fresolimumab, can neutralize all three isoforms of TGF-β (13). The bifunctional fusion protein M7824, which targets TGF-β and PD-L1, exhibited antitumor efficacy in preclinical investigations (131, 132). Moreover, the outcomes of a phase I trial involving patients with solid tumors indicated an acceptable safety profile and clinical advantages associated with M7824 (133). The efficacy of erdafitinib, an FGFR inhibitor, in overcoming resistance to fulvestrant and CDK4/6 inhibitors in MCF-7 cells has been documented (134). Additionally, AZD4547, a selective inhibitor of FGFR1/2/3, has effectively suppressed tumor cell growth, including breast cancer cells (13, 135). Furthermore, futibatinib has demonstrated the ability to inhibit the *in vitro* growth of breast cancer cells by targeting FGFR (136). Femel et al. (137) also demonstrated that the immunization of mice with extra domain-A of fibronectin resulted in a considerable decrease in both the tumor-bearing and distant metastasis rates in mouse breast cancer models. Similarly, Gagliano et al. (138) identified the fibroblast-derived p110δ subunit of phosphatidylinositol-3-OH kinase (PIK3Cδ) as a crucial mediator of TNBC. Subsequent administration of the PIK3Cδ inhibitor CAL-101 reduced tumor growth in an orthotopic breast cancer xenograft model (13).

The expression of histone deacetylase 6 (HDAC6) is frequently increased in CAFs and indicates a negative prognosis in patients with breast cancer (13). The administration of HDAC6 inhibitors reduced tumor growth rate, hindered the accumulation of bone marrow-derived monocytes and Tregs within the TME, influenced the differentiation of macrophages, and stimulated the activation of CD8- and CD4-positive T cells (139). This effect can be attributed to the upregulation of prostaglandin E2 (cyclooxygenase-2) expression by HDAC6 in CAFs, which occurs by regulating STAT3 activity (13, 139). Consequently, HDAC6 represents a promising therapeutic target for breast cancer treatment.

### 6.2 The role of CAFs in immunotherapy of breast cancer

CAFs constitute a diverse cellular population within the mammary TME and play crucial roles in modulating the immune response against tumors and influencing the efficacy of therapeutic interventions. Although CAFs directly facilitate tumor growth, metastasis, and angiogenesis, they can also contribute to the development of an immunologically inert tumor phenotype by impeding the infiltration and function of T cells or by facilitating the recruitment of other immunosuppressive cell subsets (140).

#### 6.2.1 Therapies targeting FAP molecules

Recently, there has been an increase in interest in advancing immunotherapy strategies centered around “anti-CAF” development. Notably, recent anti-CAF therapies have focused on FAP (141). The eradication of FAP+ CAFs through DNA vaccination and the use of chimeric antigen receptor T cells is a crucial adjunct to other immunotherapy approaches (39). A groundbreaking investigation demonstrated that the administration of an oral DNA-based FAP vaccine resulted in the activation of CD8+ T cells, which effectively eliminated CAFs (142). Thus, this intervention substantially enhanced the absorption of chemotherapeutic agents within the TME in multidrug-resistant mice afflicted with colon and breast cancers (142). Moreover, the FAP DNA vaccine refinement successfully circumvented immune tolerance and elicited both CD8+ and CD4+ immune reactions (39).

#### 6.2.2 Therapies targeting TGF-β and PD-L1 molecules

The activation of TGF-β in CAFs impedes T-cell infiltration and enhances the efficacy of PD-L1 antibodies in a murine model of breast cancer (143). Conversely, aberrant activation of the PD-1/PD-L1 signaling pathway is implicated in tumor immune evasion (143). Therefore, researchers have devised a therapeutic approach that concurrently targets TGF-β and PD-L1, resulting in the development of a TGF-β/PD-L1 bispecific antibody (144). This antibody has exhibited successful application in a murine breast cancer model and is currently undergoing clinical investigation (144). The inhibition of tumor cell growth can be achieved by specifically targeting the membrane-bound enzyme FAP present on the surface of CAFs (145). The administration of renin-angiotensin inhibitors can disrupt the TGF-β signaling pathway mediated by CAFs, reducing immunosuppressive response and enhancing T-cell cytotoxicity (145). Consequently, this intervention has considerable potential for improving the efficacy of immunotherapy in breast cancer treatment (145).

After the publication of the KEYNOTE-522 and -355 trials (146, 147), the Food and Drug Administration (FDA) authorized the use of pembrolizumab, a PD-L1 inhibitor, in conjunction with chemotherapy to treat patients with advanced TNBC whose tumors exhibit PD-L1 positivity. Additionally, the FDA approved pembrolizumab as a neoadjuvant treatment for high-risk early-stage TNBC, with its continued administration as a single-agent adjuvant treatment after surgical intervention (140). Notably, despite the substantial progress in the field, only a subset of patients with breast cancer undergoing immune checkpoint blockade (ICB) therapy achieve long-lasting responses, even when considering factors, such as mutational status or expression of checkpoint inhibitors (148). Therefore, exploring the factors that influence the response to ICB is imperative to devise novel therapeutic approaches that enhance the ability of the immune system to combat tumors and ultimately enhance the prognosis of patients who do not currently benefit from ICB treatment (149).

#### 6.2.3 Therapies targeting other molecules

The presence of CD10+GPR77+CAFs at moderate and high levels in breast cancer or non-small cell lung cancer tissues is correlated with resistance to chemotherapy and overall survival (OS) (150). Furthermore, the identification of a specific subset of CD10+GPR77+CAFs that promotes tumor growth, coupled with the findings that targeting CD10+GPR77+CAFs, are potential therapeutic strategies. For example, anti-GPR77 antibodies helped identify methods to inhibit breast cancer initiation and enhance the susceptibility of tumor cells to chemotherapy (150). Furthermore, the secretion of IL-6 derived from CAFs has been found to induce resistance to trastuzumab, an anti-HER2 monoclonal antibody, by activating the NF-κB, JAK/STAT3, and PI3K/AKT signaling pathways, promoting tumor stem cell proliferation, and inhibiting apoptosis. Consequently, a potential approach to overcome trastuzumab resistance in HER2-positive breast cancer could involve the combination of anti-IL-6 antibodies with inhibitors targeting these specific pathways (151).

Given the pivotal role that CAFs play in promoting immunosuppression, they present a promising avenue for augmenting breast cancer immunotherapy. The strategic targeting of CAFs is particularly important in reestablishing immune surveillance, counteracting tumor immune evasion, modulating the TME, and ultimately treating breast cancer.

### 6.3 The role of CAFs in chemotherapy and chemoresistance of breast cancer

As shown in **Figure 6**, an increasing body of research has demonstrated a strong correlation between the efficacy of chemotherapy drugs and the regulation of the DNA damage repair system (152). Several anti-tumor medications exert their therapeutic effects by inducing DNA damage in tumor cells; however, certain tumor cells can counteract the effects of DNA-damaging drugs by activating their intrinsic DNA damage repair mechanisms. Sun et al. (153) demonstrated that the upregulation of WNT16B protein expression in CAFs could damage DNA. Additionally, the secretion of the exocrine factor, SFRP2, by CAFs into the microenvironment can enhance the activation of the NF-κB signaling pathway, thereby promoting the biological activity of WNT16B (153). Consequently, this process induces resistance in breast cancer cells against mitoxantrone (153).

Doxorubicin (DOX) is classified as an anthracycline antibiotic and is utilized as both a primary treatment option for breast cancer and other tumors, as well as an adjuvant therapy for patients who have undergone breast cancer resection and exhibit axillary LN involvement. The primary cause of DOX anti-tumor therapy failure is attributed to the induction of anti-apoptotic mechanisms (154). Following apoptosis, the release of high mobility group box-1 (HMGB1) into the TME is induced (155). HMGB1 activation of CAFs in breast cancer potentially mitigate the acquired chemotherapy resistance observed in patients with breast cancer (156).

Type IV collagen, which is secreted by CAFs and serves as a constituent of the ECM, diminishes the efficacy of chemotherapy through its interaction with integrin receptors on tumor cells, thereby promoting cell adhesion and facilitating the development of drug resistance (157). Rong et al. (158) conducted a study utilizing real-time quantitative polymerase chain reaction and western blotting to examine the expression changes of IL-8 in CAFs in breast cancer before and after docetaxel chemotherapy. The findings indicated a correlation between IL-8 and resistance to breast cancer chemotherapy (158). Furthermore, the study revealed that docetaxel treatment can upregulate the expression of various genes, including *CXC2, MMP1, IL8, RARRES1, FGF1*, and *CXCR7*, in CAFs (158). This upregulation subsequently promotes the adhesion, invasion, and proliferation of MDA and MB-231 cells, ultimately leading to the development of chemotherapy resistance (158). A cellular-level investigation revealed that CAFs within the TME can diminish the chemotherapeutic efficacy of paclitaxel against breast cancer cells (128). Cui et al. (157) also discovered that MMP-1 plays a crucial role in modulating the efficacy of paclitaxel chemotherapy on breast cancer by synergistically interacting with collagen IV in CAFs.

Currently, the precise understanding of the association between fibroblasts in the TME and chemotherapy resistance remains unclear across various levels of cellular, protein, and gene analysis. Therefore, this necessitates further investigation and validation. Exploring the correlation between fibroblasts in the microenvironment of breast cancer and chemoresistance constitutes a novel research domain with substantial potential and important clinical implications for enhancing treatment effectiveness and prognosis.

### 6.4 The role of CAF in endocrine therapy and drug resistance of breast cancer

The importance of endocrine therapy is particularly pronounced in cases of hormone receptor-positive breast cancer, leading to increased interest in studying the resistance mechanism of CAFs to endocrine therapy in breast cancer. Several studies have demonstrated the importance of CAFs in developing tamoxifen resistance (159). Specifically, CAFs activate the PI3K/AKT and MAP-K/ERK pathways, contributing to this resistance (159). Additionally, Yuan et al. (160) discovered that CAFs mediated tamoxifen resistance in breast cancer cells through a G protein-coupled estrogen receptor (GPCER)-integrin β1 dependent mechanism. This process involves the upregulation of β1-integrin expression via the GPCER/GFP/ERK pathway, ultimately promoting CAF-induced EMT and subsequent tamoxifen resistance in breast cancer cells (160). Brechbuhl et al. (161) discovered that CD146 identified two separate populations of CAFs in patients with estrogen receptor-positive (ER+) breast cancer. Furthermore, they demonstrated that when MCF-7 breast cancer cells were co-cultured with CD146-CAFs, there was a reduction in the expression of ER and tamoxifen sensitivity compared with that of tumor cells co-cultured with CD146+ CAFs (161).

Another study showed that glutamine secreted by CAFs increased the survival rate and resistance to tamoxifen of breast cancer epithelial cells (162). Furthermore, CAFs play a key role in tamoxifen resistance by activating growth factor-related signaling pathways or increasing mitochondrial function to exert anti-apoptotic effects (163). In a recent study, Mao et al. (151) provided evidence indicating that CAFs play an important role in developing resistance to trastuzumab in human epidermal growth factor receptor 2 (HER2)-positive breast cancer. This resistance is believed to be mediated by various signaling pathways, including IL-6 and the activation of NF-κB, JAK/STAT3, and PI3K/AKT (151). Notably, elevated expression of PDGFRβ is linked to resistance to tamoxifen, specifically, as well as poorer prognosis, drug resistance, and increased rates of tumor recurrence in breast and prostate cancer (164). HOTAIR plays a role in augmenting ER downstream gene pathways and facilitating the processes of EMT and metastasis in breast cancer (165). Additionally, lncRNA-ROR can bind to miR205-5p, thereby instigating the EMT process (165). UCA1 has also been found to mediate tamoxifen resistance through the involvement of HIF-1α and Wnt/β-catenin (165).

Tamoxifen continues to be the most effective therapeutic intervention for ER+ breast cancer. Nevertheless, many patients encounter tamoxifen resistance accompanied by metastatic relapse, thereby presenting a substantial clinical obstacle. Therefore, to gain a deeper understanding of tamoxifen resistance within the TME, Gao et al. (166) conducted a comprehensive analysis of the microenvironment using scRNA-seq. Notably, they successfully identified a previously unrecognized subset of CAFs, known as CD63+ CAF, which plays an important role in promoting tamoxifen resistance in breast cancer (166). Furthermore, it was discovered that CD63 +CAFs release miR-22, which is abundant in the extracellular environment. This miR-22 can bind to its target, ERα, and the phosphatase and tensin homolog, resulting in the development of tamoxifen resistance in breast cancer cells (166). Importantly, the therapeutic efficacy of tamoxifen against breast cancer was improved when CD63+ CAFs were pharmacologically inhibited using CD63 neutralizing antibodies or crGd-Mir-22-sponge nanoparticles (166).

## 7 The role of CAFs in the prognosis of breast cancer

With the progress in understanding the genetic and molecular attributes of CAFs and gaining insights into the mechanisms through which CAFs facilitate tumor promotion, CAFs have emerged as potentially valuable clinical biomarkers to diagnose and prognose cancer. Currently, investigating CAFs as indicators for tumor detection has emerged as a prominent area of research. Among the markers used to assess favorable tumor prognosis are FAP, MMP-2, and MMP-9 (167, 168). The markers commonly employed in clinical practice to assess the unfavorable prognosis of tumors include α-SMA, flatfoot protein, FSP-1, vimentin, and cytoadherin C (169, 170). CAFs typically exhibit elevated levels of α-SMA, MMPs, and PDGFRα/β while displaying a diminished expression of Cav-1. In breast cancer, the presence of α-SMA-positive myofibroblasts is positively linked to tumor cell proliferation and inversely associated with both OS and recurrence-free survival (RFS) (171, 172). Furthermore, a meta-analysis has revealed an inverse correlation between α-SMA positivity in CAFs and the duration of OS and RFS in patients with breast cancer (169). In luminal breast cancer, the existence of intratumoral CAFs exhibiting a substantial upregulation of α-SMA expression (13% within the luminal breast cancer group) demonstrated a statistically significant association with poor prognosis (p=0.019) (60).

The presence of PDGFRβ in CAFs correlates with the clinical features and prognosis of individuals diagnosed with breast cancer. Moreover, a considerable correlation was found between elevated levels of PDGFRβ expression and reduced OS and disease-free survival (DFS) rates among patients with breast cancer (173). Patients exhibiting low expression of PDGFRβ can derive substantial therapeutic advantages from tamoxifen treatment (173). Additionally, Strell et al. (13) identified a specific subpopulation of fibroblasts characterized by low PDGFRα and high PDGFRβ expression, which serves as a predictive marker for increased recurrence risk in patients with ductal carcinoma *in situ*. The induction of this fibroblast subset is facilitated through contact-dependent communication between epithelial cells and fibroblasts, mediated by Jagged1 and Notch2, respectively (13). The expression of PDGFRβ in stromal cells exhibited an inverse correlation with radiation benefit, RFS, and breast cancer-specific survival (174–176). Additionally, the prognostic value of stromal PDGFRβ expression was more substantial in young and premenopausal patients with breast cancer (174, 175).

The Cox regression analysis revealed a substantial correlation between increased MMP-11 expression in CAFs and a reduced duration of RFS (177). Furthermore, GPER exhibits promise as a valuable prognostic biomarker and predictor of multidrug resistance, thereby potentially serving as a viable therapeutic target for breast cancer (178). In breast cancer, the downregulation of Cav-1 expression in CAFs has been observed, and its expression is positively associated with patient prognosis (179, 180). Notably, conflicting viewpoints have also been reported in relevant studies. Goetz et al. (181) found that Cav-1 expression was inversely correlated with prognosis in patients with breast cancer and that Cav-1 knockdown resulted in decreased fibroblast contractility. Li et al. (182) found that loss of Cav-1 expression in breast cancer cell stroma was considerably associated with poor prognosis using meta-analysis. Additionally, CAF expression was higher in metastatic axillary LNs than that in normal/reactive axillary LNs, implying that Cav-1 plays a role in breast cancer metastasis (183).

The activation of Tregs and the subsequent immunosuppressive effects mediated by FAP-positive CAFs in breast cancer occur in a manner dependent on dipeptidyl peptidase 4 (33). This mechanism has been linked to an unfavorable prognosis. Notably, a separate study revealed a positive correlation between high levels of FAP expression and extended OS and DFS in patients with invasive ductal carcinoma (167). Furthermore, the upregulation of stromal KDM2A is correlated with advanced tumor stage and unfavorable clinical outcomes among individuals diagnosed with breast cancer (184). Zeng et al. (75) discovered that elevated expression levels of CCL2 and CCL5 were associated with tumor metastasis and a negative prognosis in patients with breast cancer. Additionally, Lim et al. (185) used a tissue microarray of human breast cancer to establish a link between S100A8 expression and a poor prognosis. A positive correlation exists between the activation and expression levels of EGFR and the unfavorable prognosis of breast cancer and head and neck squamous cell carcinoma, irrespective of the administration of anticancer therapeutics (186, 187). In breast cancer, the expression of PDPN in CAFs exhibited a positive association with higher histological grade while displaying an inverse correlation with ER status, DFS, and OS (13, 188–190). The study has revealed that the presence of CD10+ GPr77+ CAFs within tumors can serve as a predictive factor for both chemotherapy response and patient survival, particularly in cases of ER- HER2- subtype and high-grade breast tumors (13). Furthermore, Bonneau et al. (191) provided evidence that CAFs of the S1 subtype contribute to the incidence of distant recurrence in early luminal breast cancer (6, 13). These important findings highlight the potential utility of CAFs as diagnostic and prognostic indicators in clinical practice.

The identification of lncRNAs as novel biomarkers has been facilitated by translational genomics and in-depth biological decoding of CAFs. For instance, the upregulation of LINC00092 by CAFs has been linked to a poorer prognosis in patients with ovarian cancer (192). Examining the entire genome transcriptome has revealed important and consistent alterations in gene expression within CAFs in breast cancer and myoepithelial populations. Furthermore, it is feasible to generate a prognostic gene signature (26-gene) capable of predicting RFS in individuals diagnosed with breast cancer (29).

Therefore, given the conflicting prognostic value of CAFs reported in several studies (161, 171), it is imperative to thoroughly address the heterogeneity of the CAF population and the diverse tumor background or progression stage. This will aid in identifying specific biomarkers that can facilitate early diagnosis, follow-up, and treatment evaluation of clinical cancer.

Summary and prospects

CAFs play an important role in mammary TME and exhibit diverse functions in the initiation and progression of breast cancer, including the facilitation of tumorigenesis, proliferation, invasion, angiogenesis, and resistance to treatment. Although some advancements have been made in investigating CAFs in breast cancer, a substantial gap remains to be clarified before their clinical application can be established. The importance of CAFs in cancer cells and tumor dynamics is increasingly evident, not only in the mammary gland, but also in other tumor entities (5). As previously stated, the importance of CAFs in cancer treatment, prognosis, and treatment outcomes has become apparent. Importantly, targeting CAFs may offer a potential solution to the persistent challenges faced in long-term breast cancer therapy, specifically distant metastasis and treatment resistance.

Currently, the primary treatment approaches for CAFs in breast cancer include the following (1): targeting the origin of CAFs by impeding their formation and inducing their reversion to a non-CAF state; however, the precise etiology of CAFs remains unknown, posing a considerable impediment to the efficacy of these therapeutic interventions (2); targeting CAFs and their secreted factors, which has been applied in clinical practice; however, there are some problems, such as lack of specific markers and low targeting efficiency, that require improvement; and (3) promoting CAFs to convert into anti-tumor molecules. The investigation of the potential of CAFs to differentiate in multiple directions and their conversion from tumor-promoting to anti-tumor or tumor-inhibiting subtypes is a key area of interest for future research in the field of tumor treatment (123). Currently, the elucidation of the origin, definition, biological heterogeneity, and other fundamental attributes of CAFs in breast cancer is yet to be clarified. Furthermore, the precise understanding of the association between CAFs within the TME and drug resistance remains elusive at the cellular, protein, and gene levels, necessitating further investigation and validation. The investigation of CAFs and drug resistance within the breast cancer microenvironment constitutes an emerging field with vast potential and substantial clinical implications for enhancing treatment efficacy and prognosis. A comprehensive investigation of CAFs will contribute to a better understanding of the intricate interplay between cancer cells and the various components of the breast cancer microenvironment, consequently paving the way for novel therapeutic approaches to managing breast cancer.

## Author contributions

JZ: Writing – original draft. HH: Conceptualization, Funding acquisition, Project administration, Supervision, Writing – review & editing.

## References

[1] Siegel RL Miller KD Jemal A. Cancer statistics, 2019. CA Cancer J Clin (2019) 69(1):7–34. doi: 10.3322/caac.21551 30620402

[2] Maajani K Jalali A Alipour S Khodadost M Tohidinik HR Yazdani K. The global and regional survival rate of women with breast cancer: A systematic review and meta-analysis. Clin Breast Cancer (2019) 19(3):165–77. doi: 10.1016/j.clbc.2019.01.006 30952546

[3] Soongsathitanon J Jamjuntra P Sumransub N Yangngam S de la Fuente M Landskron G. Crosstalk between tumor-infiltrating immune cells and cancer-associated fibroblasts in tumor growth and immunosuppression of breast cancer. J Immunol Res (2021) 2021:8840066. doi: 10.1155/2021/8840066 34337083 PMC8294979

[4] Houthuijzen JM Jonkers J. Cancer-associated fibroblasts as key regulators of the breast cancer tumor microenvironment. Cancer Metastasis Rev (2018) 37(4):577–97. doi: 10.1007/s10555-018-9768-3 30465162

[5] Elwakeel E Weigert A. Breast cancer cafs: spectrum of phenotypes and promising targeting avenues. Int J Mol Sci (2021) 22(21):11636. doi: 10.3390/ijms222111636 PMC8583860 34769066

[6] Chen X Song E. Turning foes to friends: targeting cancer-associated fibroblasts. Nat Rev Drug Discov (2019) 18(2):99–115. doi: 10.1038/s41573-018-0004-1 30470818

[7] Sahai E Astsaturov I Cukierman E DeNardo DG Egeblad M Evans RM. A framework for advancing our understanding of cancer-associated fibroblasts. Nat Rev Cancer (2020) 20(3):174–86. doi: 10.1038/s41568-019-0238-1 PMC7046529 31980749

[8] Darby IA Hewitson TD. Fibroblast differentiation in wound healing and fibrosis. Int Rev Cytol (2007) 257:143–79. doi: 10.1016/s0074-7696(07)57004-x 17280897

[9] Jun JI Lau LF. The matricellular protein ccn1 induces fibroblast senescence and restricts fibrosis in cutaneous wound healing. Nat Cell Biol (2010) 12(7):676–85. doi: 10.1038/ncb2070 PMC2919364 20526329

[10] Kalluri R Zeisberg M. Fibroblasts in cancer. Nat Rev Cancer (2006) 6(5):392–401. doi: 10.1038/nrc1877 16572188

[11] Dzobo K Dandara C. Architecture of cancer-associated fibroblasts in tumor microenvironment: mapping their origins, heterogeneity, and role in cancer therapy resistance. Omics (2020) 24(6):314–39. doi: 10.1089/omi.2020.0023 32496970

[12] Kojima Y Acar A Eaton EN Mellody KT Scheel C Ben-Porath I. Autocrine tgf-beta and stromal cell-derived factor-1 (Sdf-1) signaling drives the evolution of tumor-promoting mammary stromal myofibroblasts. Proc Natl Acad Sci USA (2010) 107(46):20009–14. doi: 10.1073/pnas.1013805107 PMC2993333 21041659

[13] Hu D Li Z Zheng B Lin X Pan Y Gong P. Cancer-associated fibroblasts in breast cancer: challenges and opportunities. Cancer Commun (Lond) (2022) 42(5):401–34. doi: 10.1002/cac2.12291 PMC9118050 35481621

[14] Ohlund D Elyada E Tuveson D. Fibroblast heterogeneity in the cancer wound. J Exp Med (2014) 211(8):1503–23. doi: 10.1084/jem.20140692 PMC4113948 25071162

[15] Gaggioli C Hooper S Hidalgo-Carcedo C Grosse R Marshall JF Harrington K. Fibroblast-led collective invasion of carcinoma cells with differing roles for rhogtpases in leading and following cells. Nat Cell Biol (2007) 9(12):1392–400. doi: 10.1038/ncb1658 18037882

[16] Liu T Zhou L Li D Andl T Zhang Y. Cancer-associated fibroblasts build and secure the tumor microenvironment. Front Cell Dev Biol (2019) 7:60. doi: 10.3389/fcell.2019.00060 31106200 PMC6492564

[17] Giorello MB Borzone FR Labovsky V Piccioni FV Chasseing NA. Cancer-associated fibroblasts in the breast tumor microenvironment. J Mammary Gland Biol Neoplasia (2021) 26(2):135–55. doi: 10.1007/s10911-020-09475-y 33398516

[18] Yang F Ning Z Ma L Liu W Shao C Shu Y. Exosomal mirnas and mirna dysregulation in cancer-associated fibroblasts. Mol Cancer (2017) 16(1):148. doi: 10.1186/s12943-017-0718-4 PMC5576273 28851377

[19] Yang SS Ma S Dou H Liu F Zhang SY Jiang C. Breast cancer-derived exosomes regulate cell invasion and metastasis in breast cancer via mir-146a to activate cancer associated fibroblasts in tumor microenvironment. Exp Cell Res (2020) 391(2):111983. doi: 10.1016/j.yexcr.2020.111983 32268136

[20] Zhu Y Dou H Liu Y Yu P Li F Wang Y. Breast cancer exosome-derived mir-425-5p induces cancer-associated fibroblast-like properties in human mammary fibroblasts by tgfβ1/ros signaling pathway. Oxid Med Cell Longev (2022) 2022:5266627. doi: 10.1155/2022/5266627 36506936 PMC9729028

[21] Li Q Lv X Han C Kong Y Dai Z Huo D. Enhancer reprogramming promotes the activation of cancer-associated fibroblasts and breast cancer metastasis. Theranostics (2022) 12(17):7491–508. doi: 10.7150/thno.75853 PMC9691365 36438487

[22] De Vincenzo A Belli S Franco P Telesca M Iaccarino I Botti G. Paracrine recruitment and activation of fibroblasts by C-myc expressing breast epithelial cells through the Igfs/Igf-1r axis. Int J Cancer (2019) 145(10):2827–39. doi: 10.1002/ijc.32613 31381136

[23] Weber CE Kothari AN Wai PY Li NY Driver J Zapf MAC. Osteopontin mediates an mzf1–tgf-B1-dependent transformation of mesenchymal stem cells into cancer-associated fibroblasts in breast cancer. Oncogene (2014) 34(37):4821–33. doi: 10.1038/onc.2014.410 PMC4476970 25531323

[24] Barth PJ Ebrahimsade S Ramaswamy A Moll R. Cd34+ Fibrocytes in invasive ductal carcinoma, ductal carcinoma in situ, and benign breast lesions. Virchows Arch (2002) 440(3):298–303. doi: 10.1007/s004280100530 11889601

[25] Iwano M Plieth D Danoff TM Xue C Okada H Neilson EG. Evidence that fibroblasts derive from epithelium during tissue fibrosis. J Clin Invest (2002) 110(3):341–50. doi: 10.1172/jci15518 PMC151091 12163453

[26] Zeisberg EM Potenta S Xie L Zeisberg M Kalluri R. Discovery of endothelial to mesenchymal transition as a source for carcinoma-associated fibroblasts. Cancer Res (2007) 67(21):10123–8. doi: 10.1158/0008-5472.Can-07-3127 17974953

[27] Jotzu C Alt E Welte G Li J Hennessy BT Devarajan E. Adipose Tissue Derived Stem Cells Differentiate into Carcinoma-Associated Fibroblast-Like Cells under the Influence of Tumor Derived Factors. Cell Oncol (Dordr) (2011) 34(1):55–67. doi: 10.1007/s13402-011-0012-1 21327615 PMC13014585

[28] Hosaka K Yang Y Seki T Fischer C Dubey O Fredlund E. Pericyte-fibroblast transition promotes tumor growth and metastasis. Proc Natl Acad Sci USA (2016) 113(38):E5618–27. doi: 10.1073/pnas.1608384113 PMC5035870 27608497

[29] Piersma B Hayward MK Weaver VM. Fibrosis and cancer: A strained relationship. Biochim Biophys Acta Rev Cancer (2020) 1873(2):188356. doi: 10.1016/j.bbcan.2020.188356 32147542 PMC7733542

[30] Madar S Goldstein I Rotter V. ‘Cancer associated fibroblasts’–more than meets the eye. Trends Mol Med (2013) 19(8):447–53. doi: 10.1016/j.molmed.2013.05.004 23769623

[31] Kobayashi H Enomoto A Woods SL Burt AD Takahashi M Worthley DL. Cancer-associated fibroblasts in gastrointestinal cancer. Nat Rev Gastroenterol Hepatol (2019) 16(5):282–95. doi: 10.1038/s41575-019-0115-0 30778141

[32] Chen Y McAndrews KM Kalluri R. Clinical and therapeutic relevance of cancer-associated fibroblasts. Nat Rev Clin Oncol (2021) 18(12):792–804. doi: 10.1038/s41571-021-00546-5 34489603 PMC8791784

[33] Costa A Kieffer Y Scholer-Dahirel A Pelon F Bourachot B Cardon M. Fibroblast heterogeneity and immunosuppressive environment in human breast cancer. Cancer Cell (2018) 33(3):463–79.e10. doi: 10.1016/j.ccell.2018.01.011 29455927

[34] Pelon F Bourachot B Kieffer Y Magagna I Mermet-Meillon F Bonnet I. Cancer-associated fibroblast heterogeneity in axillary lymph nodes drives metastases in breast cancer through complementary mechanisms. Nat Commun (2020) 11(1):404. doi: 10.1038/s41467-019-14134-w 31964880 PMC6972713

[35] Kieffer Y Hocine HR Gentric G Pelon F Bernard C Bourachot B. Single-cell analysis reveals fibroblast clusters linked to immunotherapy resistance in cancer. Cancer Discov (2020) 10(9):1330–51. doi: 10.1158/2159-8290.Cd-19-1384 32434947

[36] Salimifard S Masjedi A Hojjat-Farsangi M Ghalamfarsa G Irandoust M Azizi G. Cancer associated fibroblasts as novel promising therapeutic targets in breast cancer. Pathol Res Pract (2020) 216(5):152915. doi: 10.1016/j.prp.2020.152915 32146002

[37] Jung YY Lee YK Koo JS. Expression of cancer-associated fibroblast-related proteins in adipose stroma of breast cancer. Tumour Biol (2015) 36(11):8685–95. doi: 10.1007/s13277-015-3594-9 26044562

[38] Park SY Kim HM Koo JS. Differential expression of cancer-associated fibroblast-related proteins according to molecular subtype and stromal histology in breast cancer. Breast Cancer Res Treat (2015) 149(3):727–41. doi: 10.1007/s10549-015-3291-9 25667103

[39] Liu T Han C Wang S Fang P Ma Z Xu L. Cancer-associated fibroblasts: an emerging target of anti-cancer immunotherapy. J Hematol Oncol (2019) 12(1):86. doi: 10.1186/s13045-019-0770-1 31462327 PMC6714445

[40] Avalle L Raggi L Monteleone E Savino A Viavattene D Statello L. Stat3 induces breast cancer growth via angptl4, mmp13 and stc1 secretion by cancer associated fibroblasts. Oncogene (2022) 41(10):1456–67. doi: 10.1038/s41388-021-02172-y 35042959

[41] Houthuijzen JM de Bruijn R van der Burg E Drenth AP Wientjens E Filipovic T. Cd26-negative and cd26-positive tissue-resident fibroblasts contribute to functionally distinct caf subpopulations in breast cancer. Nat Commun (2023) 14(1):183. doi: 10.1038/s41467-023-35793-w 36635273 PMC9837080

[42] Jabbari K Cheng Q Winkelmaier G Furuta S Parvin B. Cd36(+) fibroblasts secrete protein ligands that growth-suppress triple-negative breast cancer cells while elevating adipogenic markers for a model of cancer-associated fibroblast. Int J Mol Sci (2022) 23(21):12744. doi: 10.3390/ijms232112744 PMC9654220 36361532

[43] Barone I Vircillo V Giordano C Gelsomino L Győrffy B Tarallo R. Activation of farnesoid X receptor impairs the tumor-promoting function of breast cancer-associated fibroblasts. Cancer Lett (2018) 437:89–99. doi: 10.1016/j.canlet.2018.08.026 30176263

[44] Venning FA Zornhagen KW Wullkopf L Sjölund J Rodriguez-Cupello C Kjellman P. Deciphering the temporal heterogeneity of cancer-associated fibroblast subpopulations in breast cancer. J Exp Clin Cancer Res (2021) 40(1):175. doi: 10.1186/s13046-021-01944-4 34016130 PMC8138934

[45] Roulot A Héquet D Guinebretière JM Vincent-Salomon A Lerebours F Dubot C. Tumoral heterogeneity of breast cancer. Ann Biol Clin (Paris) (2016) 74(6):653–60. doi: 10.1684/abc.2016.1192 27848916

[46] Hanahan D Weinberg RA. Hallmarks of cancer: the next generation. Cell (2011) 144(5):646–74. doi: 10.1016/j.cell.2011.02.013 21376230

[47] Junttila MR de Sauvage FJ. Influence of tumour micro-environment heterogeneity on therapeutic response. Nature (2013) 501(7467):346–54. doi: 10.1038/nature12626 24048067

[48] Li C Wu H Guo L Liu D Yang S Li S. Single-cell transcriptomics reveals cellular heterogeneity and molecular stratification of cervical cancer. Commun Biol (2022) 5(1):1208. doi: 10.1038/s42003-022-04142-w 36357663 PMC9649750

[49] Chen K Wang Q Li M Guo H Liu W Wang F. Single-cell rna-seq reveals dynamic change in tumor microenvironment during pancreatic ductal adenocarcinoma Malignant progression. EBioMedicine (2021) 66:103315. doi: 10.1016/j.ebiom.2021.103315 33819739 PMC8047497

[50] Scholer-Dahirel A Costa A Mechta-Grigoriou F. Control of cancer-associated fibroblast function by oxidative stress: A new piece in the puzzle. Cell Cycle (2013) 12(14):2169. doi: 10.4161/cc.25547 23803729 PMC3755062

[51] Ershaid N Sharon Y Doron H Raz Y Shani O Cohen N. Nlrp3 inflammasome in fibroblasts links tissue damage with inflammation in breast cancer progression and metastasis. Nat Commun (2019) 10(1):4375. doi: 10.1038/s41467-019-12370-8 31558756 PMC6763472

[52] Santolla MF Talia M Cirillo F Scordamaglia D De Rosis S Spinelli A. The ages/rage transduction signaling prompts il-8/cxcr1/2-mediated interaction between cancer-associated fibroblasts (Cafs) and breast cancer cells. Cells (2022) 11(15):2402. doi: 10.3390/cells11152402 PMC9368521 35954247

[53] Sun X Qu Q Lao Y Zhang M Yin X Zhu H. Tumor suppressor hic1 is synergistically compromised by cancer-associated fibroblasts and tumor cells through the il-6/pstat3 axis in breast cancer. BMC Cancer (2019) 19(1):1180. doi: 10.1186/s12885-019-6333-6 31795965 PMC6891969

[54] Donnarumma E Fiore D Nappa M Roscigno G Adamo A Iaboni M. Cancer-associated fibroblasts release exosomal micrornas that dictate an aggressive phenotype in breast cancer. Oncotarget (2017) 8(12):19592–608. doi: 10.18632/oncotarget.14752 PMC5386708 28121625

[55] Wang H Wei H Wang J Li L Chen A Li Z. Microrna-181d-5p-containing exosomes derived from cafs promote emt by regulating cdx2/hoxa5 in breast cancer. Mol Ther Nucleic Acids (2020) 19:654–67. doi: 10.1016/j.omtn.2019.11.024 PMC6970169 31955007

[56] Yan Z Sheng Z Zheng Y Feng R Xiao Q Shi L. Cancer-associated fibroblast-derived exosomal mir-18b promotes breast cancer invasion and metastasis by regulating tceal7. Cell Death Dis (2021) 12(12):1120. doi: 10.1038/s41419-021-04409-w 34853307 PMC8636636

[57] Chen B Sang Y Song X Zhang D Wang L Zhao W. Exosomal mir-500a-5p derived from cancer-associated fibroblasts promotes breast cancer cell proliferation and metastasis through targeting Usp28. Theranostics (2021) 11(8):3932–47. doi: 10.7150/thno.53412 PMC7914354 33664871

[58] Wu HJ Hao M Yeo SK Guan JL. Fak signaling in cancer-associated fibroblasts promotes breast cancer cell migration and metastasis by exosomal mirnas-mediated intercellular communication. Oncogene (2020) 39(12):2539–49. doi: 10.1038/s41388-020-1162-2 PMC7310603 31988451

[59] Xi C Wang J Sun H Zhang X Kang H. Retracted: loss of microrna-30e induced by extracellular vesicles from cancer-associated fibroblasts promotes breast cancer progression by binding to Cthrc1. Exp Mol Pathol (2021) 118:104586. doi: 10.1016/j.yexmp.2020.104586 33264647

[60] Muchlińska A Nagel A Popęda M Szade J Niemira M Zieliński J. Alpha-smooth muscle actin-positive cancer-associated fibroblasts secreting osteopontin promote growth of luminal breast cancer. Cell Mol Biol Lett (2022) 27(1):45. doi: 10.1186/s11658-022-00351-7 35690734 PMC9188043

[61] Suh J Kim DH Lee YH Jang JH Surh YJ. Fibroblast growth factor-2, derived from cancer-associated fibroblasts, stimulates growth and progression of human breast cancer cells via Fgfr1 signaling. Mol Carcinog (2020) 59(9):1028–40. doi: 10.1002/mc.23233 32557854

[62] Huang M Fu M Wang J Xia C Zhang H Xiong Y. Tgf-B1-activated cancer-associated fibroblasts promote breast cancer invasion, metastasis and epithelial-mesenchymal transition by autophagy or overexpression of Fap-A. Biochem Pharmacol (2021) 188:114527. doi: 10.1016/j.bcp.2021.114527 33741330

[63] Ren J Smid M Iaria J Salvatori DCF van Dam H Zhu HJ. Cancer-associated fibroblast-derived gremlin 1 promotes breast cancer progression. Breast Cancer Res (2019) 21(1):109. doi: 10.1186/s13058-019-1194-0 31533776 PMC6751614

[64] Elwakeel E Brüggemann M Wagih J Lityagina O Elewa MAF Han Y. Disruption of prostaglandin E2 signaling in cancer-associated fibroblasts limits mammary carcinoma growth but promotes metastasis. Cancer Res (2022) 82(7):1380–95. doi: 10.1158/0008-5472.Can-21-2116 35105690

[65] Chen Z Yan X Li K Ling Y Kang H. Stromal fibroblast-derived mfap5 promotes the invasion and migration of breast cancer cells via notch1/slug signaling. Clin Transl Oncol (2020) 22(4):522–31. doi: 10.1007/s12094-019-02156-1 31190277

[66] Cao Y Cao W Qiu Y Zhou Y Guo Q Gao Y. Oroxylin a suppresses actn1 expression to inactivate cancer-associated fibroblasts and restrain breast cancer metastasis. Pharmacol Res (2020) 159:104981. doi: 10.1016/j.phrs.2020.104981 32492489

[67] De Francesco EM Sims AH Maggiolini M Sotgia F Lisanti MP Clarke RB. Gper mediates the angiocrine actions induced by Igf1 through the Hif-1alpha/Vegf pathway in the breast tumor microenvironment. Breast Cancer Res (2017) 19(1):129. doi: 10.1186/s13058-017-0923-5 29212519 PMC5719673

[68] Cadamuro M Brivio S Mertens J Vismara M Moncsek A Milani C. Platelet-derived growth factor-D enables liver myofibroblasts to promote tumor lymphangiogenesis in cholangiocarcinoma. J Hepatol (2019) 70(4):700–9. doi: 10.1016/j.jhep.2018.12.004 PMC10878126 30553841

[69] De Palma M Biziato D Petrova TV. Microenvironmental regulation of tumour angiogenesis. Nat Rev Cancer (2017) 17(8):457–74. doi: 10.1038/nrc.2017.51 28706266

[70] Al-Kharashi LA Tulbah A Arafah M Eldali AM Al-Tweigeri T Aboussekhra A. High dnmt1 expression in stromal fibroblasts promotes angiogenesis and unfavorable outcome in locally advanced breast cancer patients. Front Oncol (2022) 12:877219. doi: 10.3389/fonc.2022.877219 35719957 PMC9202650

[71] Wan X Guan S Hou Y Qin Y Zeng H Yang L. Fosl2 promotes vegf-independent angiogenesis by transcriptionnally activating Wnt5a in breast cancer-associated fibroblasts. Theranostics (2021) 11(10):4975–91. doi: 10.7150/thno.55074 PMC7978317 33754039

[72] Sjoberg E Meyrath M Milde L Herrera M Lovrot J Hagerstrand D. A novel Ackr2-dependent role of fibroblast-derived Cxcl14 in epithelial-to-mesenchymal transition and metastasis of breast cancer. Clin Cancer Res (2019) 25(12):3702–17. doi: 10.1158/1078-0432.CCR-18-1294 30850359

[73] Augsten M Sjöberg E Frings O Vorrink SU Frijhoff J Olsson E. Cancer-associated fibroblasts expressing cxcl14 rely upon Nos1-derived nitric oxide signaling for their tumor-supporting properties. Cancer Res (2014) 74(11):2999–3010. doi: 10.1158/0008-5472.Can-13-2740 24710408

[74] Eiro N González L Martínez-Ordoñez A Fernandez-Garcia B González LO Cid S. Cancer-associated fibroblasts affect breast cancer cell gene expression, invasion and angiogenesis. Cell Oncol (Dordr) (2018) 41(4):369–78. doi: 10.1007/s13402-018-0371-y PMC12995208 29497991

[75] Zeng H Hou Y Zhou X Lang L Luo H Sun Y. Cancer-associated fibroblasts facilitate premetastatic niche formation through lncrna snhg5-mediated angiogenesis and vascular permeability in breast cancer. Theranostics (2022) 12(17):7351–70. doi: 10.7150/thno.74753 PMC9691361 36438499

[76] Li Z Sun C Qin Z. Metabolic reprogramming of cancer-associated fibroblasts and its effect on cancer cell reprogramming. Theranostics (2021) 11(17):8322–36. doi: 10.7150/thno.62378 PMC8343997 34373744

[77] Sun K Tang S Hou Y Xi L Chen Y Yin J. Oxidized atm-mediated glycolysis enhancement in breast cancer-associated fibroblasts contributes to tumor invasion through lactate as metabolic coupling. EBioMedicine (2019) 41:370–83. doi: 10.1016/j.ebiom.2019.02.025 PMC6442874 30799198

[78] De Jaeghere EA Denys HG De Wever O. Fibroblasts fuel immune escape in the tumor microenvironment. Trends Cancer (2019) 5(11):704–23. doi: 10.1016/j.trecan.2019.09.009 31735289

[79] Zhang J Shi Z Xu X Yu Z Mi J. The influence of microenvironment on tumor immunotherapy. FEBS J (2019) 286(21):4160–75. doi: 10.1111/febs.15028 PMC6899673 31365790

[80] Penkert J Ripperger T Schieck M Schlegelberger B Steinemann D Illig T. On metabolic reprogramming and tumor biology: A comprehensive survey of metabolism in breast cancer. Oncotarget (2016) 7(41):67626–49. doi: 10.18632/oncotarget.11759 PMC5341901 27590516

[81] Martinez-Outschoorn UE Lisanti MP Sotgia F. Catabolic cancer-associated fibroblasts transfer energy and biomass to anabolic cancer cells, fueling tumor growth. Semin Cancer Biol (2014) 25:47–60. doi: 10.1016/j.semcancer.2014.01.005 24486645

[82] Molina ML Garcia-Bernal D Martinez S Valdor R. Autophagy in the immunosuppressive perivascular microenvironment of glioblastoma. Cancers (Basel) (2019) 12(1):47–60. doi: 10.3390/cancers12010102 PMC7016956 31906065

[83] Kim SH Lee HY Jung SP Kim S Lee JE Nam SJ. Role of secreted type I collagen derived from stromal cells in two breast cancer cell lines. Oncol Lett (2014) 8(2):507–12. doi: 10.3892/ol.2014.2199 PMC4081378 25013462

[84] Dong H Diao H Zhao Y Xu H Pei S Gao J. Overexpression of matrix metalloproteinase-9 in breast cancer cell lines remarkably increases the cell Malignancy largely via activation of transforming growth factor beta/smad signalling. Cell Prolif (2019) 52(5):e12633. doi: 10.1111/cpr.12633 31264317 PMC6797518

[85] Luo H Tu G Liu Z Liu M. Cancer-associated fibroblasts: A multifaceted driver of breast cancer progression. Cancer Lett (2015) 361(2):155–63. doi: 10.1016/j.canlet.2015.02.018 25700776

[86] Wang JP Hielscher A. Fibronectin: how its aberrant expression in tumors may improve therapeutic targeting. J Cancer (2017) 8(4):674–82. doi: 10.7150/jca.16901 PMC5370511 28367247

[87] Insua-Rodriguez J Oskarsson T. The extracellular matrix in breast cancer. Adv Drug Deliv Rev (2016) 97:41–55. doi: 10.1016/j.addr.2015.12.017 26743193

[88] Erdogan B Ao M White LM Means AL Brewer BM Yang L. Cancer-associated fibroblasts promote directional cancer cell migration by aligning fibronectin. J Cell Biol (2017) 216(11):3799–816. doi: 10.1083/jcb.201704053 PMC5674895 29021221

[89] Lee KW Yeo SY Sung CO Kim SH. Twist1 is a key regulator of cancer-associated fibroblasts. Cancer Res (2015) 75(1):73–85. doi: 10.1158/0008-5472.CAN-14-0350 25368021

[90] Yu Y Xiao CH Tan LD Wang QS Li XQ Feng YM. Cancer-associated fibroblasts induce epithelial-mesenchymal transition of breast cancer cells through paracrine Tgf-beta signalling. Br J Cancer (2014) 110(3):724–32. doi: 10.1038/bjc.2013.768 PMC3915130 24335925

[91] Mishra P Banerjee D Ben-Baruch A. Chemokines at the crossroads of tumor-fibroblast interactions that promote Malignancy. J Leukoc Biol (2011) 89(1):31–9. doi: 10.1189/jlb.0310182 20628066

[92] Scheel C Weinberg RA. Cancer stem cells and epithelial–mesenchymal transition: concepts and molecular links. Semin Cancer Biol (2012) 22(5-6):396–403. doi: 10.1016/j.semcancer.2012.04.001 22554795 PMC6220425

[93] Chaffer CL Marjanovic ND Lee T Bell G Kleer CG Reinhardt F. Poised chromatin at the zeb1 promoter enables breast cancer cell plasticity and enhances tumorigenicity. Cell (2013) 154(1):61–74. doi: 10.1016/j.cell.2013.06.005 23827675 PMC4015106

[94] Scherz-Shouval R Santagata S Mendillo ML Sholl LM Ben-Aharon I Beck AH. The reprogramming of tumor stroma by hsf1 is a potent enabler of Malignancy. Cell (2014) 158(3):564–78. doi: 10.1016/j.cell.2014.05.045 PMC4249939 25083868

[95] Liu Y Geng YH Yang H Yang H Zhou YT Zhang HQ. Extracellular atp drives breast cancer cell migration and metastasis via S100a4 production by cancer cells and fibroblasts. Cancer Lett (2018) 430:1–10. doi: 10.1016/j.canlet.2018.04.043 29733962

[96] Pakravan K Mossahebi-Mohammadi M Ghazimoradi MH Cho WC Sadeghizadeh M Babashah S. Monocytes educated by cancer-associated fibroblasts secrete exosomal Mir-181a to activate akt signaling in breast cancer cells. J Transl Med (2022) 20(1):559. doi: 10.1186/s12967-022-03780-2 36463188 PMC9719191

[97] Liao D Luo Y Markowitz D Xiang R Reisfeld RA. Cancer associated fibroblasts promote tumor growth and metastasis by modulating the tumor immune microenvironment in a 4t1 murine breast cancer model. PloS One (2009) 4(11):e7965. doi: 10.1371/journal.pone.0007965 19956757 PMC2775953

[98] Ahmadzadeh M Rosenberg SA. Tgf-beta 1 attenuates the acquisition and expression of effector function by tumor antigen-specific human memory Cd8 T cells. J Immunol (2005) 174(9):5215–23. doi: 10.4049/jimmunol.174.9.5215 PMC2562293 15843517

[99] Thomas DA Massagué J. Tgf-beta directly targets cytotoxic T cell functions during tumor evasion of immune surveillance. Cancer Cell (2005) 8(5):369–80. doi: 10.1016/j.ccr.2005.10.012 16286245

[100] Cremasco V Astarita JL Grauel AL Keerthivasan S MacIsaac K Woodruff MC. Fap delineates heterogeneous and functionally divergent stromal cells in immune-excluded breast tumors. Cancer Immunol Res (2018) 6(12):1472–85. doi: 10.1158/2326-6066.CIR-18-0098 PMC6597261 30266714

[101] Pearce OMT Delaine-Smith RM Maniati E Nichols S Wang J Bohm S. Deconstruction of a metastatic tumor microenvironment reveals a common matrix response in human cancers. Cancer Discov (2018) 8(3):304–19. doi: 10.1158/2159-8290.CD-17-0284 PMC5837004 29196464

[102] Turley SJ Cremasco V Astarita JL. Immunological hallmarks of stromal cells in the tumour microenvironment. Nat Rev Immunol (2015) 15(11):669–82. doi: 10.1038/nri3902 26471778

[103] Talmadge JE Gabrilovich DI. History of myeloid-derived suppressor cells. Nat Rev Cancer (2013) 13(10):739–52. doi: 10.1038/nrc3581 PMC4358792 24060865

[104] Ugel S De Sanctis F Mandruzzato S Bronte V. Tumor-induced myeloid deviation: when myeloid-derived suppressor cells meet tumor-associated macrophages. J Clin Invest (2015) 125(9):3365–76. doi: 10.1172/jci80006 PMC4588310 26325033

[105] Gunaydin G Kesikli SA Guc D. Cancer associated fibroblasts have phenotypic and functional characteristics similar to the fibrocytes that represent a novel mdsc subset. Oncoimmunology (2015) 4(9):e1034918. doi: 10.1080/2162402x.2015.1034918 26405600 PMC4570137

[106] Qian BZ Li J Zhang H Kitamura T Zhang J Campion LR. Ccl2 recruits inflammatory monocytes to facilitate breast-tumour metastasis. Nature (2011) 475(7355):222–5. doi: 10.1038/nature10138 PMC3208506 21654748

[107] Chun E Lavoie S Michaud M Gallini CA Kim J Soucy G. Ccl2 promotes colorectal carcinogenesis by enhancing polymorphonuclear myeloid-derived suppressor cell population and function. Cell Rep (2015) 12(2):244–57. doi: 10.1016/j.celrep.2015.06.024 PMC4620029 26146082

[108] Yang X Lin Y Shi Y Li B Liu W Yin W. Fap promotes immunosuppression by cancer-associated fibroblasts in the tumor microenvironment via Stat3-Ccl2 signaling. Cancer Res (2016) 76(14):4124–35. doi: 10.1158/0008-5472.Can-15-2973 27216177

[109] Engblom C Pfirschke C Pittet MJ. The role of myeloid cells in cancer therapies. Nat Rev Cancer (2016) 16(7):447–62. doi: 10.1038/nrc.2016.54 27339708

[110] Schmid MC Varner JA. Myeloid cells in the tumor microenvironment: modulation of tumor angiogenesis and tumor inflammation. J Oncol (2010) 2010:201026. doi: 10.1155/2010/201026 20490273 PMC2871549

[111] Allaoui R Bergenfelz C Mohlin S Hagerling C Salari K Werb Z. Cancer-associated fibroblast-secreted cxcl16 attracts monocytes to promote stroma activation in triple-negative breast cancers. Nat Commun (2016) 7:13050. doi: 10.1038/ncomms13050 27725631 PMC5062608

[112] Silzle T Kreutz M Dobler MA Brockhoff G Knuechel R Kunz-Schughart LA. Tumor-associated fibroblasts recruit blood monocytes into tumor tissue. Eur J Immunol (2003) 33(5):1311–20. doi: 10.1002/eji.200323057 12731056

[113] Ma Y Hwang RF Logsdon CD Ullrich SE. Dynamic mast cell-stromal cell interactions promote growth of pancreatic cancer. Cancer Res (2013) 73(13):3927–37. doi: 10.1158/0008-5472.Can-12-4479 PMC3702652 23633481

[114] Ellem SJ Taylor RA Furic L Larsson O Frydenberg M Pook D. A pro-tumourigenic loop at the human prostate tumour interface orchestrated by oestrogen, cxcl12 and mast cell recruitment. J Pathol (2014) 234(1):86–98. doi: 10.1002/path.4386 25042571

[115] Pereira BA Lister NL Hashimoto K Teng L Flandes-Iparraguirre M Eder A. Tissue engineered human prostate microtissues reveal key role of mast cell-derived tryptase in potentiating cancer-associated fibroblast (Caf)-induced morphometric transition in vitro. Biomaterials (2019) 197:72–85. doi: 10.1016/j.biomaterials.2018.12.030 30641266

[116] Batlle E Massagué J. Transforming growth factor-B Signaling in immunity and cancer. Immunity (2019) 50(4):924–40. doi: 10.1016/j.immuni.2019.03.024 PMC7507121 30995507

[117] Donatelli SS Zhou JM Gilvary DL Eksioglu EA Chen X Cress WD. Tgf-B-inducible microrna-183 silences tumor-associated natural killer cells. Proc Natl Acad Sci USA (2014) 111(11):4203–8. doi: 10.1073/pnas.1319269111 PMC3964044 24586048

[118] Zheng S Zou Y Tang Y Yang A Liang JY Wu L. Landscape of cancer-associated fibroblasts identifies the secreted biglycan as a protumor and immunosuppressive factor in triple-negative breast cancer. Oncoimmunology (2022) 11(1):2020984. doi: 10.1080/2162402x.2021.2020984 35003899 PMC8741292

[119] Calvo F Ege N Grande-Garcia A Hooper S Jenkins RP Chaudhry SI. Mechanotransduction and yap-dependent matrix remodelling is required for the generation and maintenance of cancer-associated fibroblasts. Nat Cell Biol (2013) 15(6):637–46. doi: 10.1038/ncb2756 PMC3836234 23708000

[120] Giussani M Merlino G Cappelletti V Tagliabue E Daidone MG. Tumor-extracellular matrix interactions: identification of tools associated with breast cancer progression. Semin Cancer Biol (2015) 35:3–10. doi: 10.1016/j.semcancer.2015.09.012 26416466

[121] Yamaguchi K Hara Y Kitano I Hamamoto T Kiyomatsu K Yamasaki F. Relationship between mri findings and invasive breast cancer with podoplanin-positive cancer-associated fibroblasts. Breast Cancer (2021) 28(3):572–80. doi: 10.1007/s12282-020-01198-6 33389554

[122] Huang Y Chen L Tang Z Min Y Yu W Yang G. A novel immune and stroma related prognostic marker for invasive breast cancer in tumor microenvironment: A Tcga based study. Front Endocrinol (Lausanne) (2021) 12:774244. doi: 10.3389/fendo.2021.774244 34867821 PMC8636929

[123] Wu F Yang J Liu J Wang Y Mu J Zeng Q. Signaling pathways in cancer-associated fibroblasts and targeted therapy for cancer. Signal Transduct Target Ther (2021) 6(1):218. doi: 10.1038/s41392-021-00641-0 PMC8190181 34108441

[124] Cazet AS Hui MN Elsworth BL Wu SZ Roden D Chan CL. Targeting stromal remodeling and cancer stem cell plasticity overcomes chemoresistance in triple negative breast cancer. Nat Commun (2018) 9(1):2897. doi: 10.1038/s41467-018-05220-6 30042390 PMC6057940

[125] Chauhan VP Martin JD Liu H Lacorre DA Jain SR Kozin SV. Angiotensin inhibition enhances drug delivery and potentiates chemotherapy by decompressing tumour blood vessels. Nat Commun (2013) 4:2516. doi: 10.1038/ncomms3516 24084631 PMC3806395

[126] Coulson R Liew SH Connelly AA Yee NS Deb S Kumar B. The angiotensin receptor blocker, losartan, inhibits mammary tumor development and progression to invasive carcinoma. Oncotarget (2017) 8(12):18640–56. doi: 10.18632/oncotarget.15553 PMC5386636 28416734

[127] Sherman MH Yu RT Engle DD Ding N Atkins AR Tiriac H. Vitamin D receptor-mediated stromal reprogramming suppresses pancreatitis and enhances pancreatic cancer therapy. Cell (2014) 159(1):80–93. doi: 10.1016/j.cell.2014.08.007 25259922 PMC4177038

[128] Wilson TR Fridlyand J Yan Y Penuel E Burton L Chan E. Widespread potential for growth-factor-driven resistance to anticancer kinase inhibitors. Nature (2012) 487(7408):505–9. doi: 10.1038/nature11249 PMC3724525 22763448

[129] Takai K Le A Weaver VM Werb Z. Targeting the cancer-associated fibroblasts as a treatment in triple-negative breast cancer. Oncotarget (2016) 7(50):82889–901. doi: 10.18632/oncotarget.12658 PMC5341254 27756881

[130] Bandyopadhyay A Agyin JK Wang L Tang Y Lei X Story BM. Inhibition of pulmonary and skeletal metastasis by a transforming growth factor-beta type I receptor kinase inhibitor. Cancer Res (2006) 66(13):6714–21. doi: 10.1158/0008-5472.Can-05-3565 16818646

[131] Jochems C Tritsch SR Pellom ST Su Z Soon-Shiong P Wong HC. Analyses of functions of an anti-pd-L1/tgfβr2 bispecific fusion protein (M7824). Oncotarget (2017) 8(43):75217–31. doi: 10.18632/oncotarget.20680 PMC5650414 29088859

[132] David JM Dominguez C McCampbell KK Gulley JL Schlom J Palena C. A novel bifunctional anti-pd-L1/tgf-B Trap fusion protein (M7824) efficiently reverts mesenchymalization of human lung cancer cells. Oncoimmunology (2017) 6(10):e1349589. doi: 10.1080/2162402x.2017.1349589 29123964 PMC5665067

[133] Strauss J Heery CR Schlom J Madan RA Cao L Kang Z. Phase I trial of M7824 (Msb0011359c), a bifunctional fusion protein targeting pd-L1 and Tgfβ, in advanced solid tumors. Clin Cancer Res (2018) 24(6):1287–95. doi: 10.1158/1078-0432.Ccr-17-2653 PMC7985967 29298798

[134] Formisano L Lu Y Servetto A Hanker AB Jansen VM Bauer JA. Aberrant fgfr signaling mediates resistance to cdk4/6 inhibitors in er+ Breast cancer. Nat Commun (2019) 10(1):1373. doi: 10.1038/s41467-019-09068-2 30914635 PMC6435685

[135] Kang J Choi YJ Seo BY Jo U Park SI Kim YH. A selective fgfr inhibitor azd4547 suppresses rankl/M-csf/opg-dependent ostoclastogenesis and breast cancer growth in the metastatic bone microenvironment. Sci Rep (2019) 9(1):8726. doi: 10.1038/s41598-019-45278-w 31217507 PMC6584658

[136] Sootome H Fujita H Ito K Ochiiwa H Fujioka Y Ito K. Futibatinib is a novel irreversible fgfr 1-4 inhibitor that shows selective antitumor activity against fgfr-deregulated tumors. Cancer Res (2020) 80(22):4986–97. doi: 10.1158/0008-5472.Can-19-2568 32973082

[137] Femel J Huijbers EJ Saupe F Cedervall J Zhang L Roswall P. Therapeutic vaccination against fibronectin ed-a attenuates progression of metastatic breast cancer. Oncotarget (2014) 5(23):12418–27. doi: 10.18632/oncotarget.2628 PMC4322999 25360764

[138] Gagliano T Shah K Gargani S Lao L Alsaleem M Chen J. Pik3cδ Expression by fibroblasts promotes triple-negative breast cancer progression. J Clin Invest (2020) 130(6):3188–204. doi: 10.1172/jci128313 PMC7260014 32125284

[139] Li A Chen P Leng Y Kang J. Histone deacetylase 6 regulates the immunosuppressive properties of cancer-associated fibroblasts in breast cancer through the stat3-cox2-dependent pathway. Oncogene (2018) 37(45):5952–66. doi: 10.1038/s41388-018-0379-9 29980788

[140] Jenkins L Jungwirth U Avgustinova A Iravani M Mills A Haider S. Cancer-associated fibroblasts suppress cd8+ T-cell infiltration and confer resistance to immune-checkpoint blockade. Cancer Res (2022) 82(16):2904–17. doi: 10.1158/0008-5472.Can-21-4141 PMC9379365 35749591

[141] Ziani L Chouaib S Thiery J. Alteration of the antitumor immune response by cancer-associated fibroblasts. Front Immunol (2018) 9:414. doi: 10.3389/fimmu.2018.00414 29545811 PMC5837994

[142] Loeffler M Krüger JA Niethammer AG Reisfeld RA. Targeting tumor-associated fibroblasts improves cancer chemotherapy by increasing intratumoral drug uptake. J Clin Invest (2006) 116(7):1955–62. doi: 10.1172/jci26532 PMC1481657 16794736

[143] Gok Yavuz B Gunaydin G Gedik ME Kosemehmetoglu K Karakoc D Ozgur F. Cancer associated fibroblasts sculpt tumour microenvironment by recruiting monocytes and inducing immunosuppressive pd-1(+) tams. Sci Rep (2019) 9(1):3172. doi: 10.1038/s41598-019-39553-z 30816272 PMC6395633

[144] Lind H Gameiro SR Jochems C Donahue RN Strauss J Gulley JM. Dual targeting of tgf-beta and pd-L1 via a bifunctional anti-pd-L1/tgf-betarii agent: status of preclinical and clinical advances. J Immunother Cancer (2020) 8(1):e000433. doi: 10.1136/jitc-2019-000433 32079617 PMC7057416

[145] Chauhan VP Chen IX Tong R Ng MR Martin JD Naxerova K. Reprogramming the microenvironment with tumor-selective angiotensin blockers enhances cancer immunotherapy. Proc Natl Acad Sci USA (2019) 116(22):10674–80. doi: 10.1073/pnas.1819889116 PMC6561160 31040208

[146] Schmid P Cortes J Pusztai L McArthur H Kümmel S Bergh J. Pembrolizumab for early triple-negative breast cancer. N Engl J Med (2020) 382(9):810–21. doi: 10.1056/NEJMoa1910549 32101663

[147] Cortes J Cescon DW Rugo HS Nowecki Z Im SA Yusof MM. Pembrolizumab plus chemotherapy versus placebo plus chemotherapy for previously untreated locally recurrent inoperable or metastatic triple-negative breast cancer (Keynote-355): A randomised, placebo-controlled, double-blind, phase 3 clinical trial. Lancet (2020) 396(10265):1817–28. doi: 10.1016/S0140-6736(20)32531-9 33278935

[148] Wein L Luen SJ Savas P Salgado R Loi S. Checkpoint blockade in the treatment of breast cancer: current status and future directions. Br J Cancer (2018) 119(1):4–11. doi: 10.1038/s41416-018-0126-6 29808015 PMC6035268

[149] Keenan TE Tolaney SM. Role of immunotherapy in triple-negative breast cancer. J Natl Compr Canc Netw (2020) 18(4):479–89. doi: 10.6004/jnccn.2020.7554 32259782

[150] Su S Chen J Yao H Liu J Yu S Lao L. Cd10(+)Gpr77(+) cancer-associated fibroblasts promote cancer formation and chemoresistance by sustaining cancer stemness. Cell (2018) 172(4):841–56.e16. doi: 10.1016/j.cell.2018.01.009 29395328

[151] Mao Y Zhang Y Qu Q Zhao M Lou Y Liu J. Cancer-associated fibroblasts induce trastuzumab resistance in her2 positive breast cancer cells. Mol Biosyst (2015) 11(4):1029–40. doi: 10.1039/c4mb00710g 25648538

[152] Genois MM Paquet ER Laffitte MC Maity R Rodrigue A Ouellette M. DNA repair pathways in trypanosomatids: from DNA repair to drug resistance. Microbiol Mol Biol Rev (2014) 78(1):40–73. doi: 10.1128/mmbr.00045-13 24600040 PMC3957735

[153] Sun Y Campisi J Higano C Beer TM Porter P Coleman I. Treatment-induced damage to the tumor microenvironment promotes prostate cancer therapy resistance through wnt16b. Nat Med (2012) 18(9):1359–68. doi: 10.1038/nm.2890 PMC3677971 22863786

[154] Denel-Bobrowska M Marczak A. Structural modifications in the sugar moiety as a key to improving the anticancer effectiveness of doxorubicin. Life Sci (2017) 178:1–8. doi: 10.1016/j.lfs.2017.04.009 28431937

[155] Dong Xda E Ito N Lotze MT Demarco RA Popovic P Shand SH. High Mobility Group Box I (Hmgb1) release from tumor cells after treatment: implications for development of targeted chemoimmunotherapy. J Immunother (2007) 30(6):596–606. doi: 10.1097/CJI.0b013e31804efc76 17667523

[156] Amornsupak K Insawang T Thuwajit P OC P SA E Thuwajit C. Cancer-associated fibroblasts induce high mobility group box 1 and contribute to resistance to doxorubicin in breast cancer cells. BMC Cancer (2014) 14:955. doi: 10.1186/1471-2407-14-955 25512109 PMC4301465

[157] Cui Q Wang B Li K Sun H Hai T Zhang Y. Upregulating mmp-1 in carcinoma-associated fibroblasts reduces the efficacy of taxotere on breast cancer synergized by collagen iv. Oncol Lett (2018) 16(3):3537–44. doi: 10.3892/ol.2018.9092 PMC6096277 30127959

[158] Rong G Kang H Wang Y Hai T Sun H. Candidate markers that associate with chemotherapy resistance in breast cancer through the study on taxotere-induced damage to tumor microenvironment and gene expression profiling of carcinoma-associated fibroblasts (Cafs). PloS One (2013) 8(8):e70960. doi: 10.1371/journal.pone.0070960 23951052 PMC3738633

[159] Luo H Yang G Yu T Luo S Wu C Sun Y. Gper-mediated proliferation and estradiol production in breast cancer-associated fibroblasts. Endocr Relat Cancer (2014) 21(2):355–69. doi: 10.1530/erc-13-0237 PMC3959763 24481325

[160] Yuan J Liu M Yang L Tu G Zhu Q Chen M. Acquisition of epithelial-mesenchymal transition phenotype in the tamoxifen-resistant breast cancer cell: A new role for G protein-coupled estrogen receptor in mediating tamoxifen resistance through cancer-associated fibroblast-derived fibronectin and B1-integrin signaling pathway in tumor cells. Breast Cancer Res (2015) 17(1):69. doi: 10.1186/s13058-015-0579-y 25990368 PMC4453053

[161] Brechbuhl HM Finlay-Schultz J Yamamoto TM Gillen AE Cittelly DM Tan AC. Fibroblast subtypes regulate responsiveness of luminal breast cancer to estrogen. Clin Cancer Res (2017) 23(7):1710–21. doi: 10.1158/1078-0432.Ccr-15-2851 PMC5378660 27702820

[162] Ko YH Lin Z Flomenberg N Pestell RG Howell A Sotgia F. Glutamine fuels a vicious cycle of autophagy in the tumor stroma and oxidative mitochondrial metabolism in epithelial cancer cells: implications for preventing chemotherapy resistance. Cancer Biol Ther (2011) 12(12):1085–97. doi: 10.4161/cbt.12.12.18671 PMC3335942 22236876

[163] Martinez-Outschoorn UE Goldberg A Lin Z Ko YH Flomenberg N Wang C. Anti-estrogen resistance in breast cancer is induced by the tumor microenvironment and can be overcome by inhibiting mitochondrial function in epithelial cancer cells. Cancer Biol Ther (2011) 12(10):924–38. doi: 10.4161/cbt.12.10.17780 PMC3280908 22041887

[164] Nurmik M Ullmann P Rodriguez F Haan S Letellier E. In search of definitions: cancer-associated fibroblasts and their markers. Int J Cancer (2020) 146(4):895–905. doi: 10.1002/ijc.32193 30734283 PMC6972582

[165] Yang M Sun Y Ji H Zhang Q. Identification and validation of endocrine resistance-related and immune-related long non-coding rna (Lncrna) signatures for predicting endocrinotherapy response and prognosis in breast cancer. Ann Transl Med (2022) 10(24):1399. doi: 10.21037/atm-22-6158 36660659 PMC9843421

[166] Gao Y Li X Zeng C Liu C Hao Q Li W. Cd63(+) cancer-associated fibroblasts confer tamoxifen resistance to breast cancer cells through exosomal mir-22. Adv Sci (Weinh) (2020) 7(21):2002518. doi: 10.1002/advs.202002518 33173749 PMC7610308

[167] Ariga N Sato E Ohuchi N Nagura H Ohtani H. Stromal expression of fibroblast activation protein/seprase, a cell membrane serine proteinase and gelatinase, is associated with longer survival in patients with invasive ductal carcinoma of breast. Int J Cancer (2001) 95(1):67–72. doi: 10.1002/1097-0215(20010120)95:1<67::aid-ijc1012>3.0.co;2-u 11241314

[168] Niemiec J Adamczyk A Malecki K Ambicka A Rys J. Tumor grade and matrix metalloproteinase 2 expression in stromal fibroblasts help to stratify the high-risk group of patients with early breast cancer identified on the basis of st gallen recommendations. Clin Breast Cancer (2013) 13(2):119–28. doi: 10.1016/j.clbc.2012.12.005 23375518

[169] Hu G Xu F Zhong K Wang S Huang L Chen W. Activated tumor-infiltrating fibroblasts predict worse prognosis in breast cancer patients. J Cancer (2018) 9(20):3736–42. doi: 10.7150/jca.28054 PMC6216016 30405845

[170] Yang Z Ni W Cui C Fang L Xuan Y. Tenascin C is a prognostic determinant and potential cancer-associated fibroblasts marker for breast ductal carcinoma. Exp Mol Pathol (2017) 102(2):262–7. doi: 10.1016/j.yexmp.2017.02.012 28223108

[171] Paulsson J Micke P. Prognostic relevance of cancer-associated fibroblasts in human cancer. Semin Cancer Biol (2014) 25:61–8. doi: 10.1016/j.semcancer.2014.02.006 24560651

[172] Surowiak P Murawa D Materna V Maciejczyk A Pudelko M Ciesla S. Occurence of stromal myofibroblasts in the invasive ductal breast cancer tissue is an unfavourable prognostic factor. Anticancer Res (2007) 27(4c):2917–24. 17695471

[173] Hu G Huang L Zhong K Meng L Xu F Wang S. Pdgfr-B(+) fibroblasts deteriorate survival in human solid tumors: A meta-analysis. Aging (Albany NY) (2021) 13(10):13693–707. doi: 10.18632/aging.202952 PMC8202854 33946048

[174] Paulsson J Sjöblom T Micke P Pontén F Landberg G Heldin CH. Prognostic significance of stromal platelet-derived growth factor beta-receptor expression in human breast cancer. Am J Pathol (2009) 175(1):334–41. doi: 10.2353/ajpath.2009.081030 PMC2708819 19498003

[175] Strell C Stenmark Tullberg A Jetne Edelmann R Akslen LA Malmström P Fernö M. Prognostic and predictive impact of stroma cells defined by pdgfrb expression in early breast cancer: results from the randomized swebcg91rt trial. Breast Cancer Res Treat (2021) 187(1):45–55. doi: 10.1007/s10549-021-06136-4 33661437 PMC8062362

[176] Frings O Augsten M Tobin NP Carlson J Paulsson J Pena C. Prognostic significance in breast cancer of a gene signature capturing stromal pdgf signaling. Am J Pathol (2013) 182(6):2037–47. doi: 10.1016/j.ajpath.2013.02.018 23583284

[177] Eiro N Cid S Fernandez B Fraile M Cernea A Sanchez R. Mmp11 expression in intratumoral inflammatory cells in breast cancer. Histopathology (2019) 75(6):916–30. doi: 10.1111/his.13956 31342542

[178] Yu T Yang G Hou Y Tang X Wu C Wu XA. Cytoplasmic gper translocation in cancer-associated fibroblasts mediates camp/pka/creb/glycolytic axis to confer tumor cells with multidrug resistance. Oncogene (2017) 36(15):2131–45. doi: 10.1038/onc.2016.370 27721408

[179] Mercier I Casimiro MC Wang C Rosenberg AL Quong J Minkeu A. Human breast cancer-associated fibroblasts (Cafs) show caveolin-1 downregulation and rb tumor suppressor functional inactivation: implications for the response to hormonal therapy. Cancer Biol Ther (2008) 7(8):1212–25. doi: 10.4161/cbt.7.8.6220 PMC6688494 18458534

[180] Simpkins SA Hanby AM Holliday DL Speirs V. Clinical and functional significance of loss of caveolin-1 expression in breast cancer-associated fibroblasts. J Pathol (2012) 227(4):490–8. doi: 10.1002/path.4034 22488553

[181] Goetz JG Minguet S Navarro-Lérida I Lazcano JJ Samaniego R Calvo E. Biomechanical remodeling of the microenvironment by stromal caveolin-1 favors tumor invasion and metastasis. Cell (2011) 146(1):148–63. doi: 10.1016/j.cell.2011.05.040 PMC3244213 21729786

[182] Li X Sun J Hu S. Expression of caveolin-1 in breast cancer stroma as a potential prognostic biomarker of survival and progression: A meta-analysis. Wien Klin Wochenschr (2017) 129(15-16):558–63. doi: 10.1007/s00508-017-1173-3 28364168

[183] Scatena C Fanelli G Fanelli GN Menicagli M Aretini P Ortenzi V. New insights in the expression of stromal caveolin 1 in breast cancer spread to axillary lymph nodes. Sci Rep (2021) 11(1):2755. doi: 10.1038/s41598-021-82405-y 33531603 PMC7854652

[184] Chen JY Li CF Lai YS Hung WC. Lysine demethylase 2a expression in cancer-associated fibroblasts promotes breast tumour growth. Br J Cancer (2021) 124(2):484–93. doi: 10.1038/s41416-020-01112-z PMC7852571 33024266

[185] Lim H Koh M Jin H Bae M Lee SY Kim KM. Cancer-associated fibroblasts induce an aggressive phenotypic shift in non-malignant breast epithelial cells via interleukin-8 and S100a8. J Cell Physiol (2021) 236(10):7014–32. doi: 10.1002/jcp.30364 33748944

[186] Yoshida GJ. Regulation of heterogeneous cancer-associated fibroblasts: the molecular pathology of activated signaling pathways. J Exp Clin Cancer Res (2020) 39(1):112. doi: 10.1186/s13046-020-01611-0 PMC7296768 32546182

[187] Ciardiello F. Epidermal growth factor receptor (EGFR) as a target in cancer therapy: understanding the role of receptor expression and other molecular determinants that could influence the response to anti-EGFR drugs. Eur J Cancer (2003) 39(10):1348–54. doi: 10.1016/s0959-8049(03)00235-1 12826036

[188] Hu G Tortora G Xu F Ding Q Chen W Zhong K. Tumor-infiltrating podoplanin+ Fibroblasts predict worse outcome in solid tumors. Cell Physiol Biochem (2018) 51(3):1041–50. doi: 10.1159/000495484 30476924

[189] Pula B Jethon A Piotrowska A Gomulkiewicz A Owczarek T Calik J. Podoplanin expression by cancer-associated fibroblasts predicts poor outcome in invasive ductal breast carcinoma. Histopathology (2011) 59(6):1249–60. doi: 10.1111/j.1365-2559.2011.04060.x 22175904

[190] Schoppmann SF Berghoff A Dinhof C Jakesz R Gnant M Dubsky P. Podoplanin-expressing cancer-associated fibroblasts are associated with poor prognosis in invasive breast cancer. Breast Cancer Res Treat (2012) 134(1):237–44. doi: 10.1007/s10549-012-1984-x 22350732

[191] Bonneau C Eliès A Kieffer Y Bourachot B Ladoire S Pelon F. A subset of activated fibroblasts is associated with distant relapse in early luminal breast cancer. Breast Cancer Res (2020) 22(1):76. doi: 10.1186/s13058-020-01311-9 32665033 PMC7362513

[192] Zhao L Ji G Le X Wang C Xu L Feng M. Long noncoding rna linc00092 acts in cancer-associated fibroblasts to drive glycolysis and progression of ovarian cancer. Cancer Res (2017) 77(6):1369–82. doi: 10.1158/0008-5472.Can-16-1615 28087599

